# A protein self-assembly model guided by electrostatic and hydrophobic dipole moments

**DOI:** 10.1371/journal.pone.0216253

**Published:** 2019-04-29

**Authors:** Angel Mozo-Villarías, Enrique Querol

**Affiliations:** Institut de Biotecnologia i Biomedicina, Universitat Autònoma de Barcelona, Barcelona, Spain; Russian Academy of Medical Sciences, RUSSIAN FEDERATION

## Abstract

Protein self-assembling is studied under the light of the Biological Membrane model. To this purpose we define a simplified formulation of hydrophobic interaction energy in analogy with electrostatic energy stored in an electric dipole. Self-assembly is considered to be the result of the balanced influence of electrostatic and hydrophobic interactions, limited by steric hindrance as a consequence of the relative proximity of their components. Our analysis predicts the type of interaction that drives an assembly. We study the growth of both electrostatic and hydrophobic energies stored by a protein system as it self-assembles. Each type of assembly is studied by using two examples, PDBid 2OM3 (hydrophobic) and PDBid 3ZEE (electrostatic). Other systems are presented to show the application of our procedure. We also study the relative orientation of the monomers constituting the first dimer of a protein assembly to check whether their relative position provides the optimal interaction energy (energy minimum). It is shown that the inherent orientation of the dimers corresponds to the optimum energy (energy minimum) of assembly compatible with steric limitations. These results confirm and refine our Biological Membrane model of protein self-assembly valid for all open and closed systems.

## Introduction

It has been proposed that the configuration adopted by protein monomers when they self assemble forming large and complex structures is analogous to that adopted by a double layer of phospholipids constituting a biological membrane under the hydrophobic effect [1–3]. This configuration appears in every type of structure ranging from actin filaments and microtubules to all types of viral capsids, amyloid fibers, protein cages, etc. However, assembled protein structures seldom resemble biological membranes. The reason being, besides the obvious higher complexity and variety of proteins, the existence of other significant interactions, such as electrostatic (due to the presence of charged amino acids), hydrogen bonds, etc. In particular, charged amino acids exert attractions or repulsions on groups that are also subject to hydrophobic attractions or repulsions. In addition to these effects, spatial limitations on the monomers also have an unavoidable influence on the particular configuration any given protein assembly may adopt [4, 5]. Every self-assembled protein system is thus, the result of the nuanced contribution of all these interactions. The particular intensities and proportions of these interactions present in a given assembled protein system are dependent on the composition and fold of its constituent monomers.

In previous articles [1–3], open and closed assembled systems were studied in terms of both electric dipole and hydrophobic moment vectors and their relative orientations. Some conclusions drawn for open systems in the present article are based on that previous work on closed systems. One important conclusion derived from both biological membranes and closed assembled systems was the tendency of both the total electric and hydrophobic moments of these systems to cancel. In the present coarse-grained study we propose a quantitative refinement of our “membrane model” based on the energies, both electrostatic and hydrophobic, stored in each particular assembled system.

It is clear that hydrophobic energy is a fundamental part of the whole energy content of the complexes mentioned above, together with the electrostatic energy. Hydrophobicity as an entropic effect [6], rises as a consequence of the relative high affinity that water molecules have for each other and the subsequent exclusion of those molecules with lesser affinity. The hydrophobic force in proteins can thus be considered a consequence of the force field due to the exclusion of hydrophobic “charges” of some amino acids in aqueous media. There have been a large number of studies dealing with the effective derivation and application of combinations of force fields in order to quantitatively explain the hydrophobic effect [7–12]. In particular Lin et al. [13] and Makowski et al. [14] were successful in describing the behavior of relatively small hydrophobic molecules.

Nevertheless, in spite of all these efforts, the derivation of a practical analytical expression of a hydrophobic force field to deal with protein hydrophobicity is still lacking, probably because it is a cumbersome task, even in coarse-grain models as the one described here. Such complexity originates the need for a bold definition of a hydrophobic energy in order to facilitate a comparative treatment of both electric and hydrophobic force fields. In spite of the difference in range, the pseudo hydrophobic energy used in the present work is based on an analogy with the electrostatic potential (see Methods). It is reasonable to assume that the hydrophobic energy used in this work is a function of the actual hydrophobic energy holding a system together. The description of protein self-assembly in terms of the simultaneous action of both electrostatic and hydrophobic energies should help in the understanding of any type of complex protein association.

This work is centered on open systems, that is systems for which there are no predetermined number of elements. These electrostatic and hydrophobic energies are computed and expressed in arbitrary units. The main idea is to show that these interactions, based on the membrane model, drive monomers to assembly in all the systems reported in the PDB. It is after satisfying these forces, when the detailed specific shorter-range interactions (hydrogen bonds and others) can be established.

A large number of self-assembled systems have been crystallized and this number continues to grow. This article describes in detail two of the most characteristic and complex assemblies, PDBid 2OM3 [15] and PDBid 3ZEE [16]. These two systems have been chosen due to their complexity since they encompass all the features that characterize this type of analysis and serve for the description of other assemblies added in the S1 Appendix.

## Materials and methods

### Assembled protein systems

Coordinates of open self-assembled systems were obtained from the PDB (Protein Data Bank). Two open self-assembled protein systems were analyzed in terms of their energy content: hydrophobically driven assembly of tobacco mosaic virus PDBid 2OM3 [15] and electrostatically driven assembly of the Par-3 N-Terminal Domain of the atypical protein-kinase C complex, PDBid 3ZEE [16]. They were chosen for detailed analysis for being representatives of the two types of driving energies studied and also for being two of the most complex systems. Other systems obtained from the PDB database are deployed in S1 Appendix: linear assemblies: 1M8Q, 3G37, 4APW and 2M4J; amyloid assemblies: 3HYD, 2M5N 2M4J, 5O3L 2M4J, 2LNQ, 6CU7, 2LMN and 2RNM; complex and helicoidal assemblies: 2HIL, 3J4F, 5SYC and 3J9O.

All these systems are characterized by not having a pre-determined number of elements. In some cases they are dynamic systems that follow polymerization-depolymerization cycles.

### Energy computations

This methodology uses the concept of dipole moment for both electrostatic charges and hydrophobic “charges” in a protein. These hydrophobic charges are the normalized hydrophobic indices attributed to the amino acids. Diverse authors have published several tables with such indices and the Eisenberg scale [17] is employed here. In this scale positive hydrophobicity amino acids are those commonly known as hydrophobic amino acids and those with negative hydrophobicity are the hydrophilic amino acids. We use the term hydrophobic charge by analogy of treatment with the electric charges.

The fact that in most proteins, the total electrostatic charge (or the total hydrophobic charge) Q, is not zero, obliges the substitution of the exact dipole moment definition, **P** = ∑Q_i_. **r**_i_, for a pseudo dipole moment **P** = Q^+^. (**c**^+^–**c**^–^), where Q^+^ is the total positive charge (either electrostatic or hydrophobic), **c**^**+**^ and **c**^**−**^are the positive and negative centroids of the charges (either electrostatic or hydrophobic). Only in the case of neutral proteins, both definitions coincide. For a detailed description of these magnitudes see [18]. Results obtained by using alternative definitions of dipole moments, such as **P** = Q^−^. (**c**^+^–**c**^–^) or the exact definition, **P** = ∑Q_i_. **r**_i_, differed from those obtained by the use of **P** = Q^+^. (**c**^+^–**c**^–^), as expected. Nevertheless, the behavior of the variations of the moments did not depend on the specific definition, as long as definitions are not mixed. The preference for using the positive charges, Q^+^ in hydrophobic dipole moments is derived from the interest in hydrophobicity. These pseudo dipole moments depend on the positive charge involved, on one hand and on the distance between the positive and negative centroids, on the other. These centroid distances were found to be in the range of 1 to 10 Å.

In spite of the need to use these pseudo moments, we use here the classical theory of electrostatic dipole interaction in order to calculate their interaction energy.

The energy ***enD*** stored by two interacting electric dipoles is given by the well-known expression:

$$enD=-\frac{3\left(u_{r}\cdot D_{1}\right)\left(u_{r}\cdot D_{2}\right)-D_{1}\cdot D_{2}}{4\pi \varepsilon_{0}r^{3}}$$ (Eq 1)

where **r** is the distance vector between the two dipoles of moments **D**_**1**_ and **D**_**2**_, and **u**_**r**_ its unit vector. Dots within the equation denote scalar product of vectors (dot product). In our computations, factor 4πε_0_ is taken to be 1 and thus ***enD*** units are arbitrary.

Hydrophobic moments interact, in our force field approximation in an analogous form as the electrostatic dipole vectors but with opposite affinities. Hydrophobic charges of the same sign attract each other, whereas charges of opposite sign repel each other. As the range of hydrophobic interactions lies on distances somewhat shorter than the electrostatic ones, an inverse exponential variation instead of an inverse potential was used. Nevertheless, we find that the issue of the range of intensities of these hydrophobic interactions is of no particular relevance to our results since in principle, it only involves a scale factor with no influence on the fact that the hydrophobic force may be attractive or repulsive. We thus define a pseudo hydrophobic interaction energy ***enH*** between two hydrophobic dipoles, **H**_**1**_ and **H**_**2**_, in analogy with the electrostatic case, by the expression:

$$enH=+\frac{3\left(u_{r}\cdot H_{1}\right)\left(u_{r}\cdot H_{2}\right)-H_{1}\cdot H_{2}}{ke^{r}}$$ (Eq 2)

where ***k*** is an arbitrary constant that is also taken as 1. Note the plus sign preceding the expression for ***enH*** in contrast with the minus sign that appears in the expression for the electrostatic case (Eq 1). This positive sign arises from the different affinities that hydrophobic charges have with respect to the electrostatic case, as mentioned.

Using these formulas, for the interacting dipoles (both electric and hydrophobic), pseudo energies ***enH*** and ***enD*** were computed for all the pairs of elements constituting the systems described above. They were thus represented in arbitrary units as a function of N, number of elements in each system. The need to express these pseudo energies in arbitrary units prevents any attempt to make any kind of comparison between them. Their use is then restricted to the assessment of whether the attractive energy that drives and holds a given system is hydrophobic or electrostatic.

The computation of the energy stored in the system when a third element is added to a dimer, involves the addition of energies computed for pairs 1–2, 1–3 and 2–3, as obtained from Eqs 1 or 2. This procedure is repeated as new more elements are added to the system.

### Simulation of vector rotations

**Fig 1:**
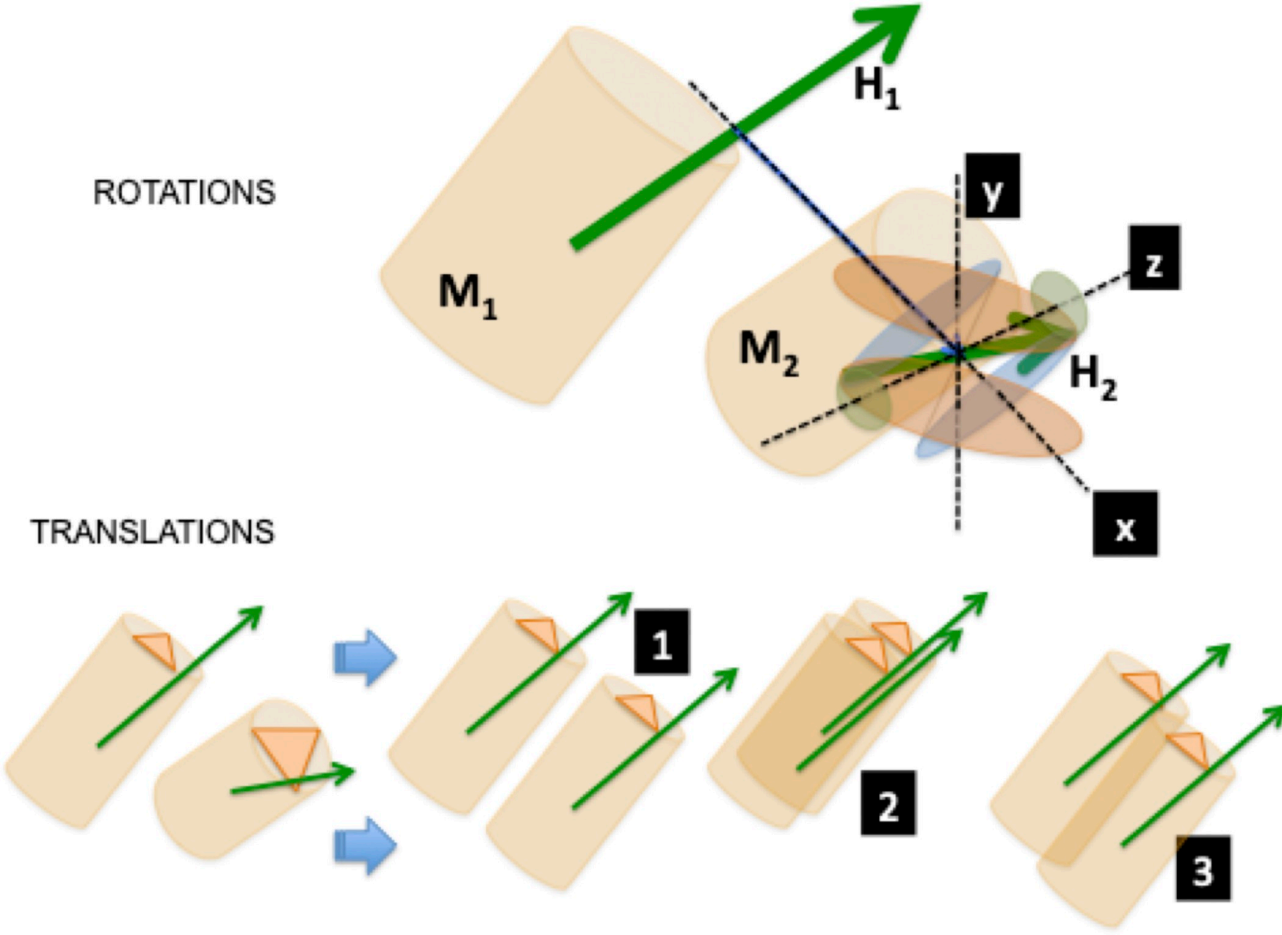
Simulation of rotations and translations of a monomer (M_2_) with respect to its neighbor (M_1_) in an assembly. The monomers have hydrophobic moment vectors **H**_**1**_ and **H**_**2**_. **x, y** and **z** labels denote the three axis of rotation. The distance vector between **H**_**1**_ and **H**_**2**_ defines the **x**–axis. Vector **H**_**2**_ rotation around this axis is visualized in the pale blue ellipses. The **y–**axis is defined by the perpendicular formed by the **x**–axis and the original vector **H**_**2**_. Rotation of **H**_**2**_ around this axis is viewed in the reddish ellipses. The **z**–axis is defined by the perpendicular direction to both **x**–axis and **y**–axis, viewed in the greenish ellipses. The same criteria are used to define the electric dipole moments rotations. Three translations are defined. In translation 1, monomers are positioned parallel side-by-side. Translation 2, monomers are positioned parallel one on top of the other. Translation 3, monomers are positioned in a combination of the former two.

It was found necessary to check whether the native addition of monomer “n+1” to monomer “n” in a given assembly, results in a conformation of optimal energy (energy minimum). For this purpose, rotations of an added monomer (monomer “2”) relative to a previous monomer (monomer “1”) in the three directions of space, were simulated and the resulting energies, ***enH*** and ***enD***, were computed for each simulated rotation by using Eqs 1 or 2. Fig 1, shows schematically this approach in which a monomer M_1_ is interacting with monomer M_2_ in order to form the first dimer of an assembly.

Both monomers have their **H** vectors (**H**_**1**_ and **H**_**2**_) and their **D** vectors (not drawn for simplicity). Both energies ***enD*** and ***enH*** are computed by means of Eqs 1 and 2 and then **H**_**2**_ (or **D**_**2**_) are rotated in three orthogonal directions, with respect to **H**_**1**_ in steps of 10°. These directions are: rotation around the **x**–axis as the joining distance vector of **H**_**1**_ and **H**_**2**_; rotation around the **y**–axis as the direction perpendicular to both the plane formed by the **x**–axis and vector **H**_**2**_; rotation around the **z**–axis as the direction perpendicular to both **x**–axis and **y**–axis. For each simulated rotation angle, ***enH*** is computed. The same procedure is applied to electric dipole moments **D**_**1**_ and **D**_**2**_, and ***enD*** is computed. It should be noted that rotations performed for the **H**_**2**_ vector are independent of those performed for the **D**_**2**_ vector.

### Simulation of vector translations

Not only different relative rotational alternatives between two monomers need to be checked out. Translational alternatives were also assayed following the criterion that these alternative positions should not further separate the monomers since separations would imply fast decrease of either ***enH*** or ***enD***.

Three alternative translations were assayed, as shown in Fig 1. First, a translation resulting with both monomers parallel to each other side-by side. Second, a translation to a top-down parallel position. Third, a combination of both translations, leaving both monomers in a staggered parallel position. Fig 1B illustrates the three translations schematically. Note that for each new simulated translation position, new rotations were simulated.

## Results

### 1. Hydrophobic-driven system. PDBid 2OM3

**Fig 2:**
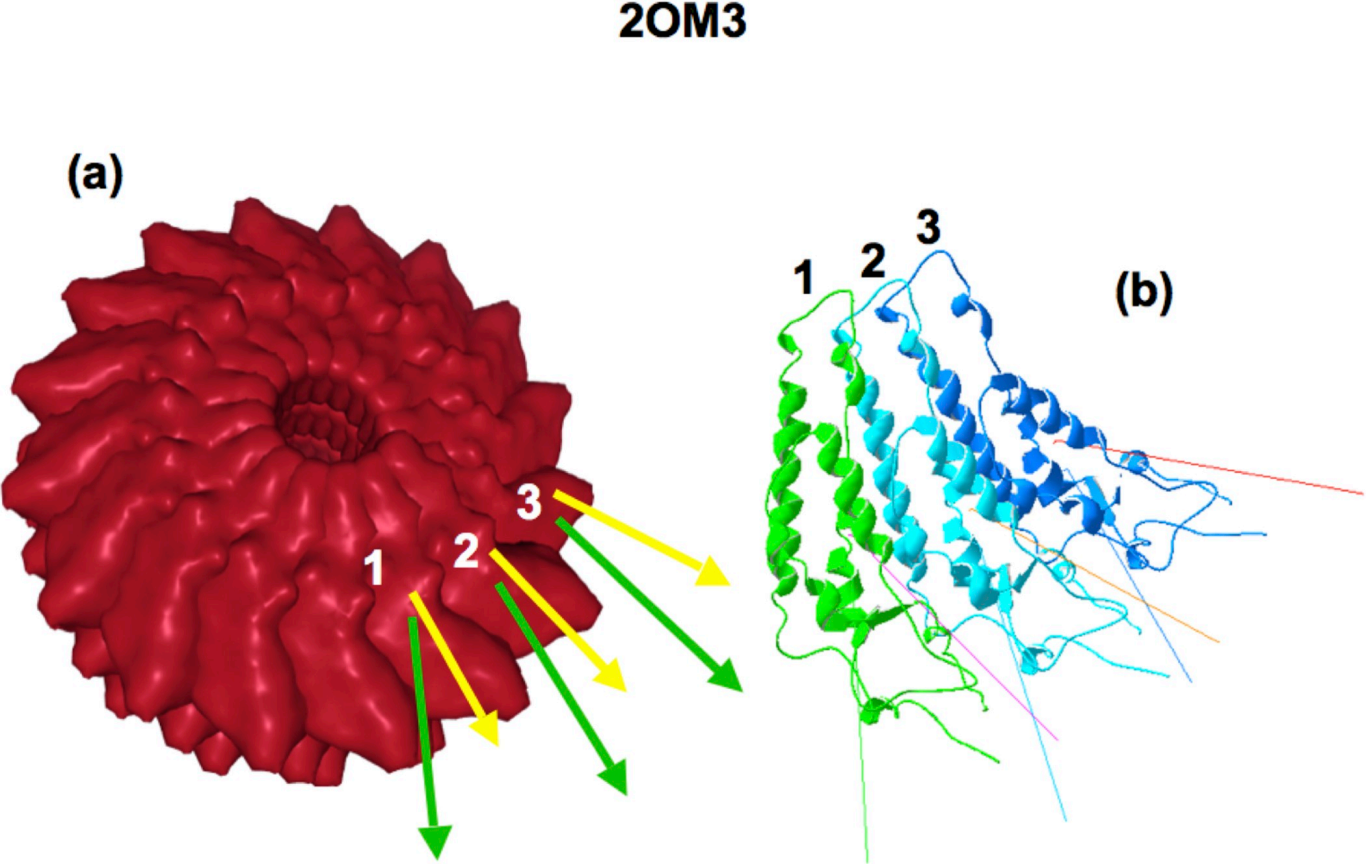
(a) Assembly of TMV capsid, PDBid 2OM3 (Source: PDB homepage), showing the **H** (green) and **D** (yellow) vectors of the first two monomers. (b) The first three elements of 2OM3. Note that the slight triangular shape of these elements makes the arrangement form a circle.

Sachse et al. [15] have recently revised the structure of the Tobacco Mosaic Virus (Fig 2).

This system consists in the side-by-side addition of basic monomers forming a spiral, as can be schematically seen in Fig 2A. The slight triangular shape that the monomers adopt in the pseudo-plane of the assembly (Fig 2B), tends to arrange them in a circle as they add up side-by-side in the complex. Moreover, since steric constraints do not allow for a perfect planar alignment of the monomers, the circle of the 16 monomers cannot exactly close on itself and thus the growth must continue on a different plane giving rise to an helicoid. This is accomplished when a new monomer is added slightly above the previous one, generating a small pitch (around 10° on average).

The hydrophobic and electrostatic energies ***enH*** and ***enD***, of several consecutive pairs of monomers were computed for 26 pairs using Eqs 2 and 1 respectively. For ***enH*** a mean value of –2.34 ± 0.25 (in arbitrary units, a.u.) was found. The error corresponds to around ±10% of the mean value. Similarly, for ***enD***, a value of +2.01 ± 0.29a.u. (±14.4%) was obtained. Although the energy values for ***enH*** y ***enD*** cannot be compared with each other, the signs of these energies clearly show that the assembly of this system is driven by hydrophobic interactions against electrostatic forces that appear to be repulsive in this model.

**Fig 3:**
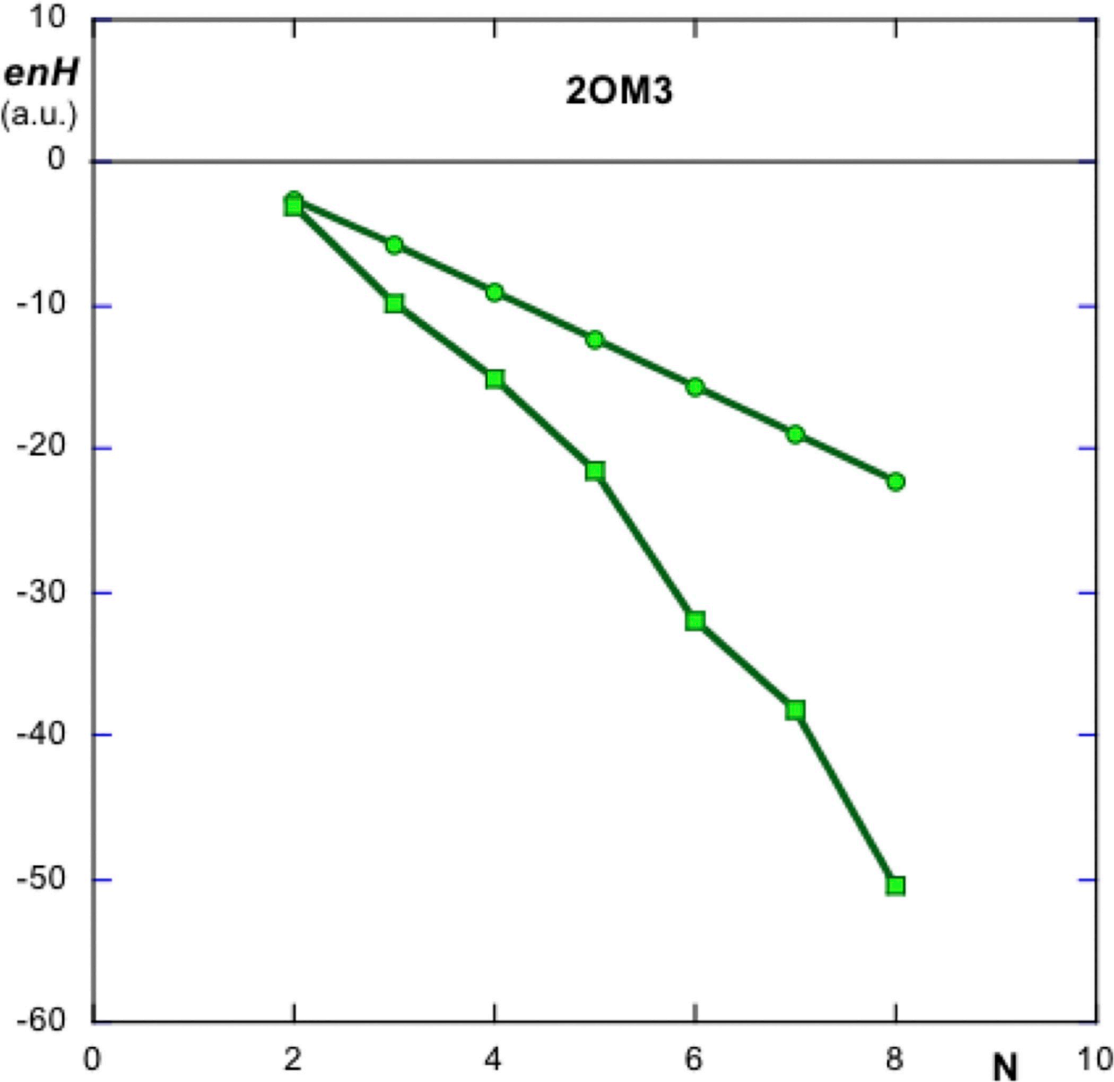
Evolution of the hydrophobic energy *enH* with N, number of added monomers. Circles refer to energies of growth as new elements are added to the system starting from the first element listed in PDBid 2OM3. Squares show the variation of ***enH*** with the number of added monomers surrounding an inner element (no.18 in this case). Similar variations of ***enD*** vs N show a growing positive linear trend (data not shown). This assembly is thus hydrophobically driven. The different slope in both curves is due to the different distances that the monomers have between them in both cases.

Fig 3 shows the variation of energy ***enH*** with N, number of elements, as they are successively added to the system. Due to the fact that the assembly grows as a spiral, it was found illustrative to compute the energy stored in the system as new monomers are added around a given element. In this case it was chosen element 18 in the PDB set of coordinates. Both variations are linear, as expected. The assembly energy computed around an inside element shows a sharper slope since the elements used in the computation are closer to the initial one and among themselves.

**Fig 4:**
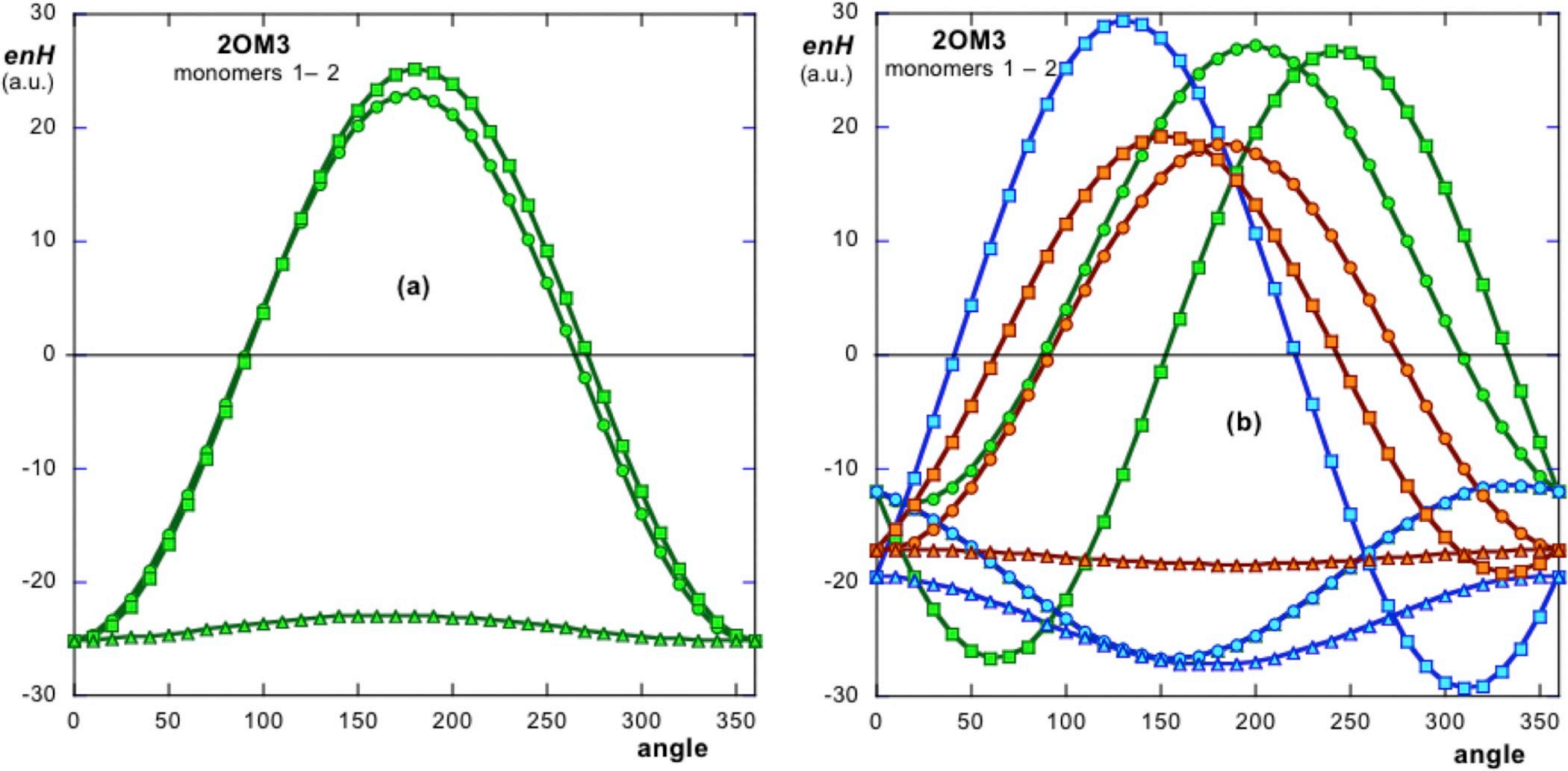
(a) Simulation of rotations of **H**_**2**_ (hydrophobic moment of monomer 2) with respect to monomer 1. Circles, squares and triangles represent rotations in the **x-**, **y-** and **z-** directions respectively. The ***enH*** curves have all their minima (attractive energies) at 0° rotation (native position). (b) Simulation of three possible translations of monomer 2 with respect to monomer 1. Note that once any translation is done, new rotation simulations (for the new translated positions) are performed. Green curves correspond to a perfect side-by-side arrangement in which the respective H vectors are parallel. Blue curves represent a translation in which both triangular monomers are positioned one on top of the other. Orange curves correspond to a combination of the former arrangements. All these alternative arrangements show less than optimal values of ***enH***.

It is necessary to check whether the energy ***enH*** obtained with Eq 2 for any pair of consecutive elements in the assembly, corresponds to a minimum, if the assembly is to be a stable structure. To this purpose we simulated rotations in all directions of space of one monomer relative to its immediate neighbor and the corresponding energy ***enH*** was computed. Fig 4A shows the variation of ***enH*** as a function of the relative orientation of the simulated rotation angle in the three directions of space (in steps of 10°), as described in Methods. It is easy to see that the native orientation of the monomers (0° rotation) corresponds to the minimum of energy in the three directions of space.

In order to further check whether the simulation of the relative translations of monomer 2 relative to monomer 1 could also show minima in their energy distributions, three different relative translational positions of the monomers were simulated. For each translation performed, new rotations were simulated and the respective angular distributions of their energies were computed (Fig 4B). These translations were designed as follows. Firstly, monomer 2 in a given dimer was made to adopt a position exactly parallel to monomer 1 (green angular distribution curves in Fig 4B).

A second dimer was created by positioning monomer 2 on top of monomer 1 (blue distribution curves in Fig 4B). A third arrangement was obtained by combining the two former arrangements (orange angular distributions in Fig 4B). Comparison of Fig 4B with 4a reveals that the native relative position of monomer 2 with respect to monomer 1 (angle 0°) yields the optimum energy to the system since curves in Fig 4B show less negative values at 0° rotations.

### 2. Electrostatic-driven system. PDBid 3ZEE

**Fig 5:**
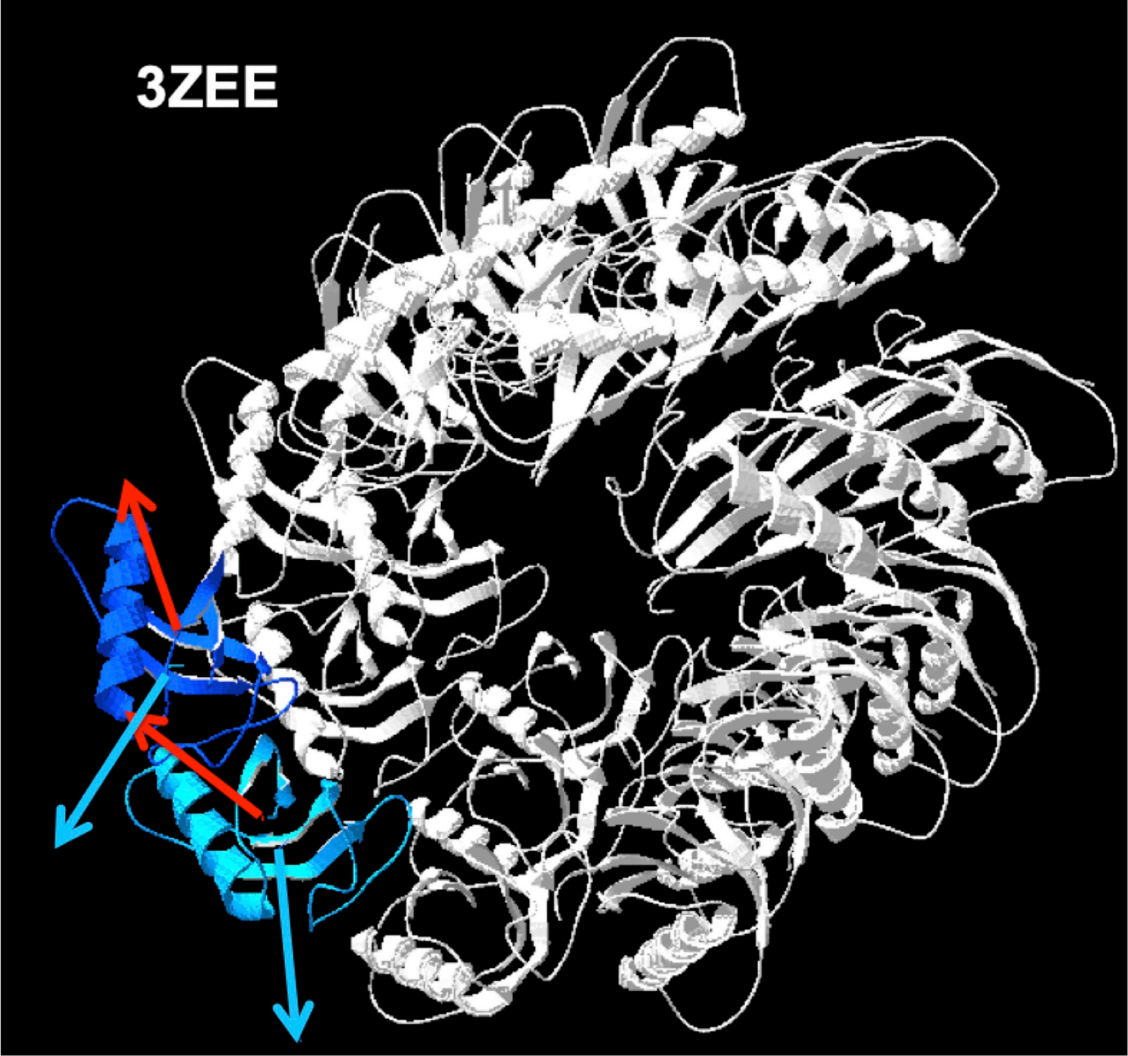
Representation of 25 subunits of the multimodular scaffold protein Par-3, PDBid 3ZEE [16]. Blue subunits were used for rotation simulations. Blue arrows are **H** vectors and red arrows are **D** vectors.

Another example of self-assembled tubular structure is the Par-3 N-Terminal Domain as reported by Zhang et al. [16]. This complex is formed by a growing number of monomers, whose frustrated lateral self-assembling forces the system to adopt a skewed configuration, ending up in a helical structure, similar to the former case. Fig 5 shows 20 of these elements.

Application of Eqs 1 and 2 to consecutive pairs of these elements yield negative (attractive) values for the energies ***enD***: –31.79 ± 3.98 a.u. (±27%) and positive (repulsive) values for ***enH***: +0.225 ± 0.009 a.u (±4%). This is indication that this assembly is electrostatically driven, in agreement with results obtained by Zhang et al. [16].

**Fig 6:**
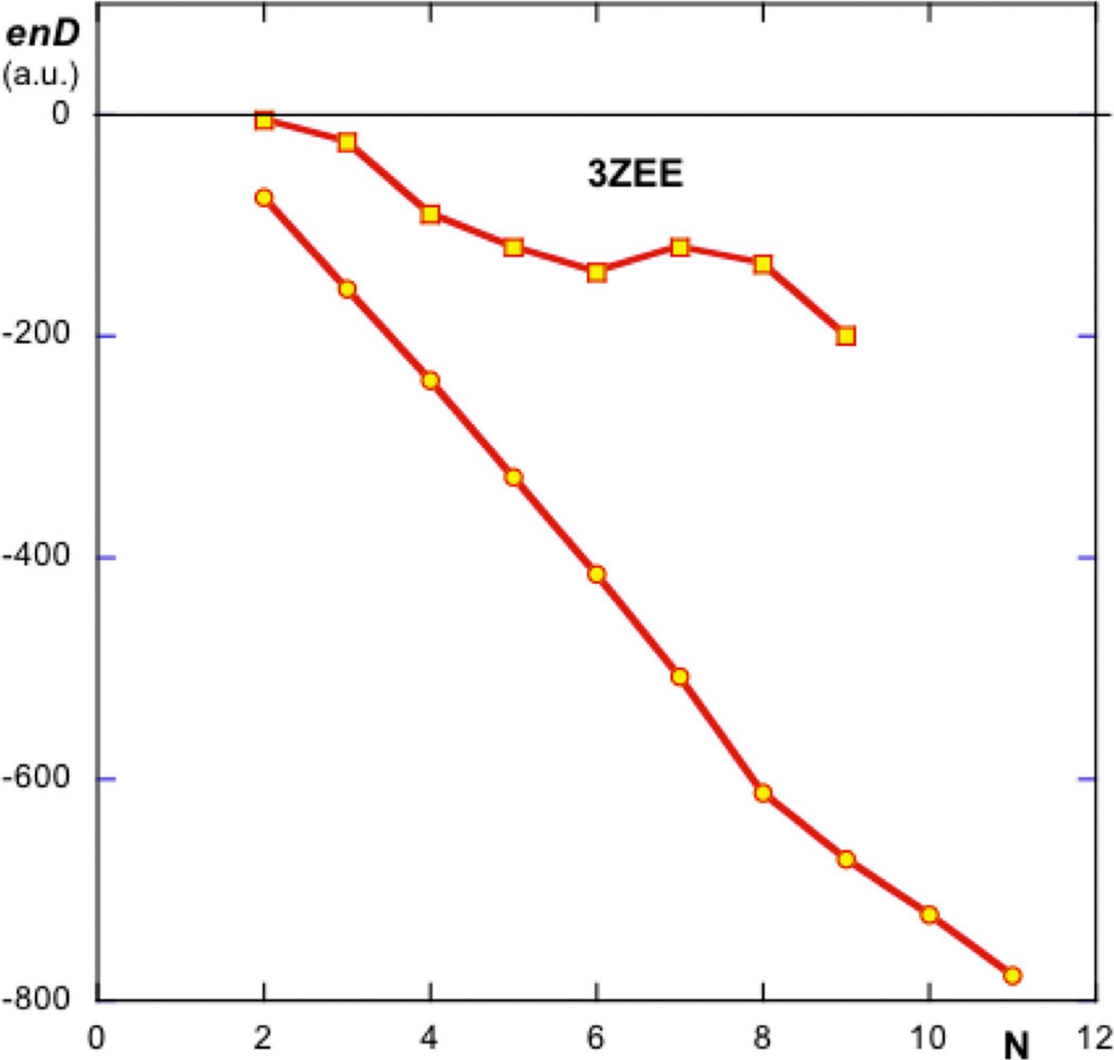
Plot of the variation of energy *enD* as a function of N as new monomers are added to the ensemble. Circles, linear addition of monomers from the first element reported in PDBid 3ZEE. Squares, variation of ***enD*** as a function of the growing number of monomers surrounding element 11. From these variations it is deduced that the assembling of this system is electrically driven against hydrophobic forces.

Following the same criterion as in the case of PDBid 2OM3 for ***enH***, the variations of ***enD*** vs. N, number of elements, are plotted both for the linear growing system and for the growing influence of surrounding monomers on a given internal monomer (no. 11 in this case). Fig 6 shows these variations. As in the case of PDBid 2OM3 for ***enH***, the variations of ***enD*** are approximately linear but with different slopes since they involve computation of energies of elements with different proximities among them.

**Fig 7:**
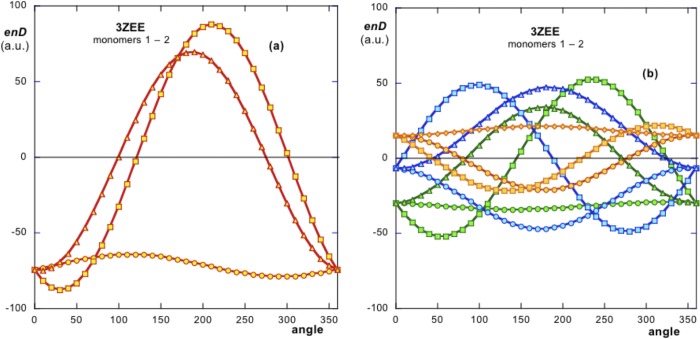
(a) shows the simulation of the variation of the energy ***enD*** under rotations in the 3D space. The three curves show that the energy minima are off from the native position. For **x-** and **y-**axis these minima are at –60°, 30° rotations respectively. It is assumed that the native orientation off the energy minimum positions is due to steric hindrances limitations on the monomers or further hydrophobic repulsions. (b) Translational simulations: green curves, translation 1; orange, translation 2; blue, translation 3 (see Methods).

In order to check whether the conformation of this structure is optimal, simulations of rotations of monomer 2 with respect to monomer 1 (see blue monomers in Fig 5), were carried out. The angular distributions obtained show values of ***enD*** that could lead, in principle to more stable conformations (Fig 7A).

Rotations of the dipole moment **D**_**2**_ around its **x**- and **y**- axis show that their ***enD*** minima do not exactly lie on the 0° angle (native orientation). For **x**-axis, the energy minimum lays around 300° (or –60°), although the variation of ***enD*** around this axis is relatively insensitive to angle. In the case of **y-**axis the energy minimum is about 30° off the native position, although the difference of this value with that of the native orientation is only marginal. These angular deviations are attributable to steric limitations. That is, these small rotations would avoid steric clashes between the monomers, so the energy levels adopted are only close to the optimal minima. Such clashes of monomers 1 and 2, are plausible visualizing Fig 5. Also further repulsive action of ***enH*** for these positions should be considered since rotations of **D**_**2**_ also implies rotations of **H**_**2**_. In spite of this, in the native configuration, the native values of ***enD*** are only marginally higher than those of the optimal minima.

Translations of monomer 2 with respect to monomer 1 were assayed following similar criteria as those applied in PDBid 2OM3 (Fig 7B). One translation consists of a side-by-side arrangement of monomers 1 and 2 in order for the monomers to lie exactly parallel to each other. The second translation consists of a top-down arrangement of the two monomers. A third translation consists of a combination of the above. Although it is necessary to stress that ***enD*** obtained from different systems cannot be compared, values of ***enD*** from the system can be compared to those that appear in Fig 7A and 7B since they all belong to different arrangements of the same system. Such comparison shows that native values of ***enD*** (0° in Fig 7A) are more negative than those in the simulations (Fig 7B). Also, minima of the energy for these translational simulated positions, would imply larger rotations.

## Discussion

We have derived a useful and simplified version of pseudo hydrophobic energy based on the use of hydrophobic moment vectors. This coarse-grained energetic model is under all aspects extraordinarily simple since it reduces large sets of atoms to electric and hydrophobic centroids where the bulk charges (electric and hydrophobic) reside. In this model the interaction of these hydrophobic “charges” (i.e. hydrophobic indices), has been rationalized in semi quantitative and empirical terms in analogy to the electrostatic energy with different power laws. Whether the energy holding the system together is attractive or repulsive depends on the type of interaction that the dipole moments exert on each other. It is also important to stress at this point that this model does not attempt to describe the kinetics of formation of these complexes but only deals with their final states.

Hydrophobic moments have been a resourceful tool for describing the hydrophobic properties of proteins, since they are easy to compute and have been shown to provide a number of applications [1–3, 12, 17–21]. The present work is based on two main pillars: the use of hydrophobic moments of proteins, as described in Methods, and the analogy of interactions of these hydrophobic moments similar to those of electric dipole moments (although with different power laws). The more crucial of these two hypotheses is the use of hydrophobic moments since they determine the sign of the hydrophobic energy, ***enH***, stored in an assembled system.

Within a protein, the permanent spacial distribution of different hydrophobicity “charges”, derived from its 3D structure, implies a force between them, and consequently, a permanent hydrophobic dipole moment can be considered. A hydrophobic dipole can interact with other hyprophobic dipoles, the same as in a biological membrane.

These concepts allow for the relatively simple study of the interactions between dipoles. Both electric and hydrophobic dipoles help in orienting monomers towards each other. Once this orientation is reached, specific local bonds are formed.

As for the other hypothesis, whether the chosen power law is similar to that of the simple electrostatic case or a different one, the resulting energy, although obviously numerically different, will maintain the same sign. Thus, it is important to note that the resulting energy ***enH*** cannot be compared to the electric energy ***enD*** and hence, the need to express both in arbitrary units.

In spite of these limitations, the model (biological membrane and pseudo hydrophobic energy) is successful in describing all self-assembled system since no system has shown an exception so far. With this method, hydrophobic energy (either attractive or repulsive) cannot be known in absolute terms, but a qualitative characterization about its variations or tendencies can be gathered for any system. Note once more that the ***enH*** values obtained for a given system cannot be compared to those of a different system since their values are structure dependent.

For one thing, it is possible to predict whether a system is either electrostatically or hydrophobically driven. In the cases described in this work (see also S1 Appendix), the energetic behavior of these systems is compatible with results reported by the authors describing them. Moreover, the method allows for the easy computation of energies in simulated alternative conformations by translating or rotating the orientation of an added monomer relative to the previous one. In all cases it has been shown that the inherent conformation corresponds to the energy minimum compatible with steric hindrances. It should be noted that these simulations are done for ***enD*** and ***enH*** independently of each other since the present model does not include a direct relationship between the relative orientations of the dipole moment vectors **H** and **D** (angle **H^D**) of the constituting monomers. It is easy to see why this angular relationship is important in determining which of the two interactions is to prevail in the complex. For example, systems in which **H** and **D** tend to be in the same direction, it is obvious that the interaction holding the structure is expected to be hydrophobic (favorable) against the electrical interaction (unfavorable). By contrast, in systems where **H** and **D** are perpendicular, the resulting assembly should easily be favored by both hydrophobic and electric interactions, provided that the growth of the assembly produces a certain degree or rotation of the monomers. Generally, **H** and **D** vectors of a protein may have any relative orientation in which case it is necessary to resort to our membrane model to figure out which of the two forces will prevail in the assembly process. By rotating (or translating) one monomer with respect to the other in a dimer, it is possible to predict the optimum configuration that these elements will adopt in an assembly, provided that steric hindrances are also considered. The analysis of other systems, as reported in S1 Appendix, backs up to all these considerations since no exceptions have been found to these conclusions.

We add a final comment regarding the potential predictability of the stability of systems based on uncommon cases in which both ***enH*** and ***enD*** energies are negative, that is, attractive. Such is the case of the hyperthermostable SP1 boiling stable protein, PDBid 1TR0. This system shows attractive values of both ***enH*** and ***enD*** in agreement with its reported high thermal stability [22].

## Conclusions

1.—In this article the biological membrane model of self-assembly is studied, refined and semi-empirically confirmed. The model works for any self-assembling protein system regardless of the complexity of the system. This model thus describes a general principle.

2.—According to this model, based on electric dipole and hydrophobic moments, hydrophobic charges of equal sign attract each other. Charges of opposite sign repel each other. This obliges hydrophobic moments to align with each other, whereas electric dipole moments tend to counter-align.

3.—This model considers a simplified form of hydrophobic energy in analogy with the electrostatic energy related to the interaction of electric dipole moments.

4.—Most assemblies do not resemble biological membranes due to the particular proportion of electrostatic and hydrophobic energies stored in the systems (plus the steric hindrances), which force the monomers to fit in, adopting slanted configurations.

5.—Most systems get their stability of assembly with one type of interaction (either electrostatic or hydrophobic) against the other. However, a few systems combine both types of attractive energies in some parts of their ensembles.

## Supporting information

S1 Appendix (DOCX)

S1 Fig Actin polymerization [23]. (a) Cartoon rendering of six assembled actin monomers, each with its **H** and **D** vectors from PDBid 1M8Q. Note the **H** vectors (long arrows) tend to align with the filament axis, whereas the **D** vectors (short arrows) rotate perpendicular to the axis. The polymerization is electrostatically driven: <***enD***> = –1.997 ± 0.029a.u. (±1.4%); <***enH***> = 0.042 ± 0.002a.u. (±4.7%). (b) Plot of the variation of ***enD*** with the number of components N of the assembled complex. (c) Simulation of variations of ***enD*** when the **D** vectors of the added monomer are rotated in the three orthogonal directions in space from their original positions. Circles, squares and triangles correspond to rotations in the **x-**, **y-** and **z**-axis respectively, as described in Fig 1 in the main text. Note in (c) that rotations around the **y**-axis, even more negative (and thus attractive) energies than the native values appear at relative high angles. These angular values however, are not accessible due to steric hindrances.
(TIF)

S2 Fig (a) Cryo-Electron structure of filamentous actin in the presence of phosphate according to Murakami et al., [24]. Blue and red arrows are **H** and **D** vectors respectively of some individual monomers in the polymer. Like in other actin polymers, **H** vectors tend to align with the polymer axis, while **D** vectors rotate perpendicularly to the axis of growth. (b) Averaging the energies of all the dimers reported: <***enD***> = –2.36 ± 0.055a.u. (±2.3%), the assembly is electrostatically driven. However, averaging ***enH*** energies of dimers a value of <***enH***> = –0.101 ± 0.021a.u. (±21%) is obtained. This means that dimers are drawn together by both electrostatic and hydrophobic forces. However, as seen in the inset of (b), from N ≥ 3, growth proceeds electrostatically only. (c) Energy distribution of ***enD*** when monomer P is rotated with respect to monomer O in the three directions of space. As in other cases, steric hindrances do not allow for perfectly symmetrical angular distributions.
(TIF)

S3 Fig (a) A peculiar actin-like polymer described by Popp et al. [25], is formed by two strands (1–2). Blue and red arrows represent **H** and **D** vectors. Each strand is the result of the addition of protofilaments ABCD… and EFG…; (b) Formation of protofilament ABCD is formed electrostatically: <***enD***> = –9.79±0.37 a.u., <***enH***> = 0.213±0.039 a.u (±3.8% and ±18.3% error respectively). Protofilaments are added hydrophobically to form a strand. Although they do not appear in the Fig, strands are added in opposite directions electrostatically to form a fiber. (c) Rotation simulations of monomer B with respect to A. Near optimum ***enD*** for y-axis rotations (45°) and relative insensitive variation in the x-axis are due to steric limitations in the arrangement as seen in other systems.
(TIF)

S4 Fig M13 filamentous bacteriophage capsid, PDBid 2MJZ. This structure was obtained combining magic-angle spinning NMR and Rosetta modeling [26]: Five α-helices associate as a close system to serve as an element of growth in a linear addition in this system. (a) Cartoon picture in which each bluish tone represents an added pentamer to the system. **H** vectors are drawn in colors matching their corresponding pentamers and **D** vectors are all colored in red. All these vectors have been drawn with a separation between them to better visualization although they are all coincident in direction. Computation of energy was carried out at two levels. First, the formation of the basic pentamer of each stage followed by the subsequent analysis of the addition of pentamers. (b) Variations of ***enD*** with N, number of elements in the assembly of one pentamer. The assembly of the pentamer is hydrophobically driven: <***enH***> = –43.6 ± 0.01a.u. (±0.01%); <***enD***> = 1860 ± 33.2.1a.u. (±1.8%). (c) Variations of ***enD*** under simulated rotations of the peptides forming each pentamer. Rotations around the y-axis show a minimum at –60° meaning that possible clashes prevent reaching the energy minimum. Rotations around the z-axis show a minimum at –160° although the energy variation is not as sensitive to rotations as in the other axis. (d) Variation of ***enH*** with N, number of pentamers added in the system as a whole. This addition is electrostatically driven. Simulation of rotations of a given pentamer with respect to its neighbor: <***enD***> = –11091 ± 4323a.u. (39%); <***enH***> = 249.9 ± 49.1a.u. (19.6%). (e) Variations of ***enH*** with rotated angle in the three direction of space. Given the symmetry present in this system, rotations around the **x**-axis leave ***enH*** of the system unaltered. The native value of ***enD*** is the optimal electrostatic energy.
(TIF)

S5 Fig (a) Association of the **LVEALYL** fragment of insulin, leading to an amyloidosis-like self-assembly (PDBid 3HYD) as reported by Ivanova et al. [27]. This is a very simple system since no electric dipole moments are present in this system.(b) Quasi-linear variation of ***enH*** with N, number of assembled elements. The system is hydrophobically driven: <***enH***> = –2621.2 ± 13.5 a.u (±0.5% standard error). (c) ***enH*** variations under rotation simulations clearly show that the native angle (0°) formed by two adjacent monomers, i.e. two blue arrows or two green arrows in (a), leads to the minimum of ***enH*** with very little restrictions.
(TIF)

S6 Fig (a) Assembly of the basic **YTIAALLSPYS** peptide (PDBid 2M5N) related to Alzheimer disease [28]. This peptide also serves for the formation of other more complex filaments. As in the case of PDBid 3HYD, the absence of electrostatic negatively charged amino acids makes this growth particularly illustrative as an example of the membrane model since it does not include electric dipole moment. The system is hydrophobically driven: <***enH***> = –7453 ± 585.2a.u (±7.8% error); (b) And (c) follow the same codes as in former Figs. Note that a rotation of peptide B with respect to A around both **x-** and **y-**axis are quasi identical, whereas rotation around the **z-**axis leaves the energy unchanged since it is a rotation around its own axis.
(TIF)

S7 Fig (a) Stacks of tau filaments in opposite orientations reported by Fitzpatrick et al. [29]. Blue and red arrows are **H** and **D** vectors respectively. (b) and (c) like in former Figs. The stacking is hydrophobically driven: <***enH***> = –0.302 ± 3.4x10^-6^a.u. (±0.001%); <***enD***> = 0.607 ± 5.5x10^-5^a.u. (±0.01%). Like in other similar cases, the monomeric peptides form stable stacks in a very symmetrical structure. These stacks form filaments by the dual joining of two stacks orienting their **H** vectors in opposite directions.
(TIF)

S8 Fig Assembly of the Aß-amyloid fibril PDB id: 2M4J [30]. (a) Association of three stacks of Aß_1–40_ fibrils. Green and red arrows denote the individual **H** and **D** vectors of each single peptide. The assembly of each stack is hydrophobically driven: <***enH***> = –6863 ± 405.2a.u; <***enD***> = 3634 ± 213.2a.u (±5.9% and ±5.8% error respectively). (b) Simulation of rotations of peptide D with respect to peptide A. (c) Simulation of rotations of the stack ADG with respect to stack BEH. As can be seen, the association of stacks is also hydrophobic. The quasi-perfect symmetry of these energy curves reflects the perfect geometrical arrangement of this association.
(TIF)

S9 Fig (a) Antiparallel array of ß-amyloid fragments (with D23N mutation) as reported by Qiang et al. [31]. (b) Variation of energy ***enH*** of the assembly as a function of N, number of elements. It was found that this assembly is hydrophobically driven: <***enH***> = –55.56 ± 4.30 a.u. (±7.7%); <***enD***> = 2040 ± 974 a.u. (±47.7%) for all the dimers computed. Certain variability in the shape and relative orientation of the individual peptides is observed in (a). This translates in a concomitant variability in the relative magnitude and direction of their individual **H** and **D** vectors. It was found necessary to compute the total energy ***enH*** following the sequence order A–B–C–D–E–F–G–H (green circles) and compare the result with the total ***enH*** energy following the reverse order (blue squares). Note that in the first case, for N = 3, ***enH*** shows a positive (repulsion) value. This is attributed to a certain “noise” originated by the lack of perfect regularity in the arrangement. This abnormality changes its position when the energy growth is computed backwards, that is, H-G-F-… (c) The angular distributions of ***enH*** when monomer C is rotated vs. monomer B, show optimal energies very near to the native configuration.
(TIF)

S10 Fig Alpha synuclein fibril. (a) Set of two stacks of α-synuclein peptides facing each other (source: PDB homepage) as described by Li et al. [32]. Blue arrows represent H vectors of the first elements in the stack and red arrows their D vectors. This assembly is hydrophobically driven: <***enH***> = –894.6 ± 6.3a.u. (±0.7%); <***enD***> = 1008.5 ± 4.4a.u. (±0.4%). e. (b) Variation of ***enH*** with the number of elements in both stacks, N. These energies were computed by adding the elements following this sequence B-D-A-G-I on one hand and C-E-F-H-J on the other hand. Both stacks attract each other by hydrophobic energy (data not shown). (c) Effect of rotations of **H** vector of element D with respect to that of element B. It can be seen that hydrophobic energy holds the system assembled at the optimum orientation as is in most cases of perfect geometrical arrangement.
(TIF)

S11 Fig (a) 2 stacks of beta-amyloid fibrils in opposite orientations (1 and 2) reported by Paravastu et al. [33]. Green and blue arrows represent the **H** vectors of the individual peptides. Red and orange vectors, their **D** vectors. The energy that maintains each stack is electrostatic: <***enD***> = –663.9 ± 159.0a.u. (±24.0%); <***enH***> = 694.9 ± 234.1a.u. (±33.7%). (b) Variation of ***enH*** with N corresponding to stack 1. Stack 2 shows a similar level of variability or noise that is attributable to the variability in shape and relative orientation of the single monomers. (c) Angular variation of ***enH*** and ***enD*** under rotation of element B with respect to element A. The variability of orientations of the **H** and **D** vectors shown in the assembly (±30°) is reflected in (c) by the fact that peptides A and B are not set at their optimal energy, especially for rotations in the **y**-axis. (d) The energy linking both stacks is hydrophobic.
(TIF)

S12 Fig (a) Prion fibrils arranged parallel according to Wasmer et al. [34]. Arrows in cold colors: individual **H** vectors of the peptides. Red arrows: **D** vectors. (b) and (c) Variations like in former Figs. Due to the unstructured tails displayed by each peptide, and the concomitant dispersion in the magnitude and direction of their **H** vectors, the system is noisy in the sense of having a notable variability in its ***enH*** values: <***enH*** > = –680.0 ± 340.7a.u. (±50%); <***enD***> = 295.8 ± 39.6 a.u. (±13.4%). It is interesting to note here that this stacked structure can be associated with other similar stacks as described by Smaoui et al. [35]. These authors show that these filaments are formed by three associated stacks arranged at a relative orientation of 120° with respect to each other (see PDBid 2M4J).
(TIF)

S13 Fig (a) Spiraling assembly by three-helix bundles from type IV pili of inner membrane bacteria as described by Craig et al. [36], obtained from Cryo-Electron Microscopy experiments. Some **H** vectors (A, B and D subunits) are depicted (black arrows). (b) The variation of energy ***enH*** with N shows that the assembling of this system is hydrophobically driven: <***enH***> = –0.704 ± 0.079a.u. (±11.2%); <***enD***> = 0.204 ± 0.006a.u. (±2.9%). (c) Angular distribution of energies when subunit B is rotated with respect to subunit A, showing optimal orientation from perfectly symmetrical energy distributions.
(TIF)

S14 Fig (a) Protein capsid of HIV-1 virus, reported by Zhao et al. [37]. The basic element of growth of this system is a hexamer. The first three hexamers (A-B-C) have been colored and their **H** vectors are displayed as pale blue arrows. (b) Variations of ***enH*** for the growing assembly (circles) or growth around an inner element, 13 (squares). The system is hydrophobically driven: ***<enH***> = –(2.3 ± 0.25) x10^-3^a.u. (±10.8%); <***enD***> = 0.861 ± 0.087a.u. (±10.1%). (c) Simulation of rotations of element B with respect to element A. The configuration of this system is very close at its optimal energy.
(TIF)

S15 Fig (a) Growing microtubule reported by Kellogg et al. [38]. The first four elements (tubulin dimers) have been cultured for clarity. Green and red arrows represent **H** and **D** vectors respectively. Although the basic element in this system is a tubulin heterodimer (i.e. yellow in (a)), according to these authors, the growth proceeds by the addition of a dimer of dimers (i.e. yellow and orange). This is the only system found in which both ***enH*** and ***enD*** are attractive energies: <***enH***> = –0.135 ± 0.018a.u. (±13.3%); <***enD***> = –1.475 ± 0.601a.u. (±41%). (b) and (c) plot the variations of ***enD*** and ***enH*** as new elements are added to the tubule. (d) and (e) Representations of rotations of **D** vectors (d) and **H** vectors (e) as described in Methods. Native energies are only near their optima especially those rotations around the **y**-axis. It is considered that this lack of symmetries in these distributions is responsible for the appearance of helicity, giving rise to the formation of the tubule as a result.
(TIF)

S16 Fig (a) Reconstruction of the structure of Type VI secretion system by Cryo-EM by Nazarov et al. [39]. It is composed by the stacking of rings each formed by six heterodimers and resembling (each ring) a crown. The heterodimers are designed as “AB”, “CD”, etc. The stacking proceeds by rotating the next ring 60° around the axis of growth with respect to the previous one. (a) shows four of these rings: (1) pale blue, (2) dark blue, (3) green and (4) purple. Thin lines design the direction of **H** and **D** vectors and the axis growth as well. The formation of the ring is electrostatic: <***enD***> = –2.532 ± 0.158a.u. (±34.5%); <***enH***> = 0.159 ± 0.040a.u. (±25.1%) The stacking of rings is also electrostatic: <***enD***> = –0.223 ± 0.077a.u. (±6.2%); <***enH***> = 0.187 ± 0.014a.u. (±7.5%). (b) Double plot of the variation of electrostatic energy ***enD*** vs. N in the formation of one of the rings (circles) and the stacking (squares). (c) and (d) Plots of the variations of ***enD*** for the ring formation and the stacking of rings respectively.
(TIF)

## Abbreviations

D, H: Electric dipole and hydrophobic moment vectors, respectively
*enH*: Hydrophobic pseudo energy
*enD*: Electrostatic energy
<…>: Average value
N: Number of components in an assembled system
Q: Total electrostatic or hydrophobic charge
Q_i_: Individual electrostatic or hydrophobic charge
Q^+^: Total positive electrostatic or hydrophobic charge
