# Supplementary material for: A protein self-assembly model guided by electrostatic and hydrophobic dipole moments
Source: PLoS One. 2019 Apr 29;14(4):e0216253. doi: 10.1371/journal.pone.0216253 (PMC6488083; doi:10.1371/journal.pone.0216253)
Supplement: S1 Appendix — (DOCX) [file pone.0216253.s001.docx]

**S1 Appendix to Supporting Information**

In the Supporting Information section, 16 examples of open systems are analyzed to further illustrate the conclusions drawn in the main text. With some exceptions, each system is presented with three panels.

Panels (a) show schematic renditions of the system, drawn from their PDB coordinates or by using images displayed in the PDB homepage. Superimposed to these images, some of their **H** and **D** vectors have also been added. For convenience, **H** vectors have been drawn as longer arrows than those of **D** vectors in order to facilitate their vision.

Panels (b) show the variations of the attractive energy that holds the system stable, with N, number of elements added to the system. In cases where this energy is hydrophobic (***enH***), this variation is plotted in green. If the attractive energy is electrostatic, ***enD***, its variation with N is plotted in red. In most cases these variation vs. N show almost perfect linear fits. Nevertheless, some systems show “noisy” variations due to variations in shapes and relative orientations along monomers in a given system. In such cases, plots of ***enH***–N or ***enD***–N are fitted to straight lines with variable degrees of accuracy.

In order to determine whether a given system is hydrophobically or electrostatically driven, computations of these energies were done for all the dimers within this system by using eqs. 1 and 2. Averages <***enH***> and <***enD***> were taken from all the dimers and presented in each figure caption, both in arbitrary units (a.u.) as reported in the main text, followed by an error estimate. The significant elements in these computations are i) the sign of the energy and ii) the percentage of error in each averaging process.

Panels (c) show the simulation of rotations of monomer n+1 with respect to monomer n in a given system, as described in Methods. Energy variations (whether ***enH*** or ***enD***) with the rotated angle are coded as follows: around **x-**axis, red; around **y-**axis, green; around **z-**axis, blue.

In the rotation simulations, whether ***enH*** or ***enD***, variations are concerned, it should be remembered that 0º rotation corresponds to the native configuration. In some systems, the angular energy distributions show that their native structures are just on or very near their minima at 0º. Others show their minima only near 0º for rotations in one or several rotation axis. Others systems show their minima far from 0º but with relative small variation from values at 0º. And when ***enH*** (or ***enD***) energy is responsible for the assembly, a combination of steric limitations and/or the repulsive action of ***enD*** (or ***enH***), prevent the system from adopting the absolute energy minimum configuration.

Systems PDBid 2MJZ and PDBid 3J9O are formed by elements, which are closed systems on their own. In these cases, the closed system, that serves as monomer for the supra open system, has also been analyzed.
