# Supplementary figures and images for: A protein self-assembly model guided by electrostatic and hydrophobic dipole moments
Source: PLoS One. 2019 Apr 29;14(4):e0216253. doi: 10.1371/journal.pone.0216253 (PMC6488083; doi:10.1371/journal.pone.0216253)

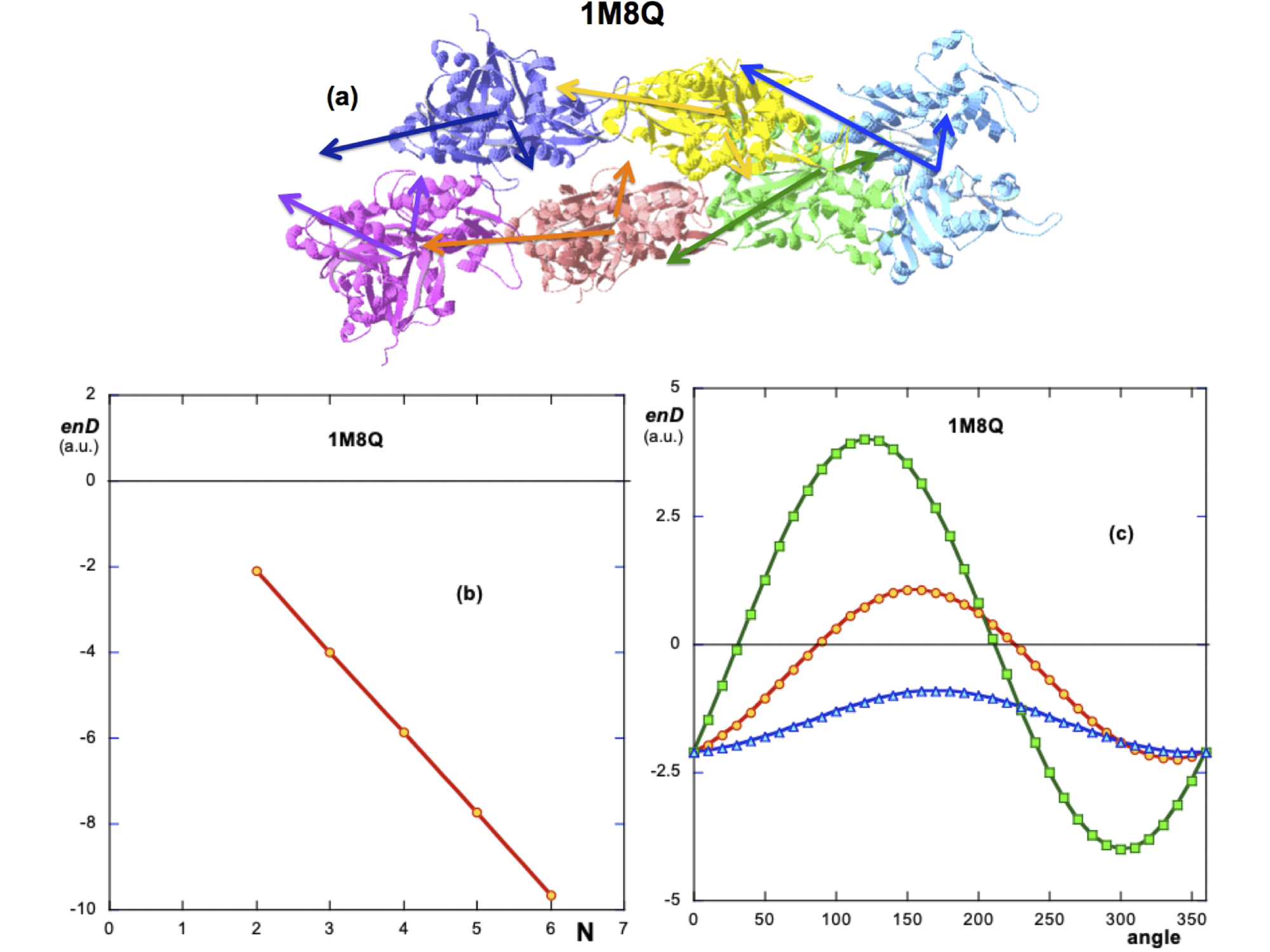

Supplement: S1 Fig — (a) Cartoon rendering of six assembled actin monomers, each with its H and D vectors from PDBid 1M8Q. Note the H vectors (long arrows) tend to align with the filament axis, whereas the D vectors (short arrows) rotate perpendicular to the axis. The polymerization is electrostatically driven: = –1.997 ± 0.029a.u. (±1.4%); = 0.042 ± 0.002a.u. (±4.7%). (b) Plot of the variation of enD with the number of components N of the assembled complex. (c) Simulation of variations of enD when the D vectors of the added monomer are rotated in the three orthogonal directions in space from their original positions. Circles, squares and triangles correspond to rotations in the x-, y- and z-axis respectively, as described in Fig 1 in the main text. Note in (c) that rotations around the y-axis, even more negative (and thus attractive) energies than the native values appear at relative high angles. These angular values however, are not accessible due to steric hindrances. (TIF) [file pone.0216253.s002.tif]

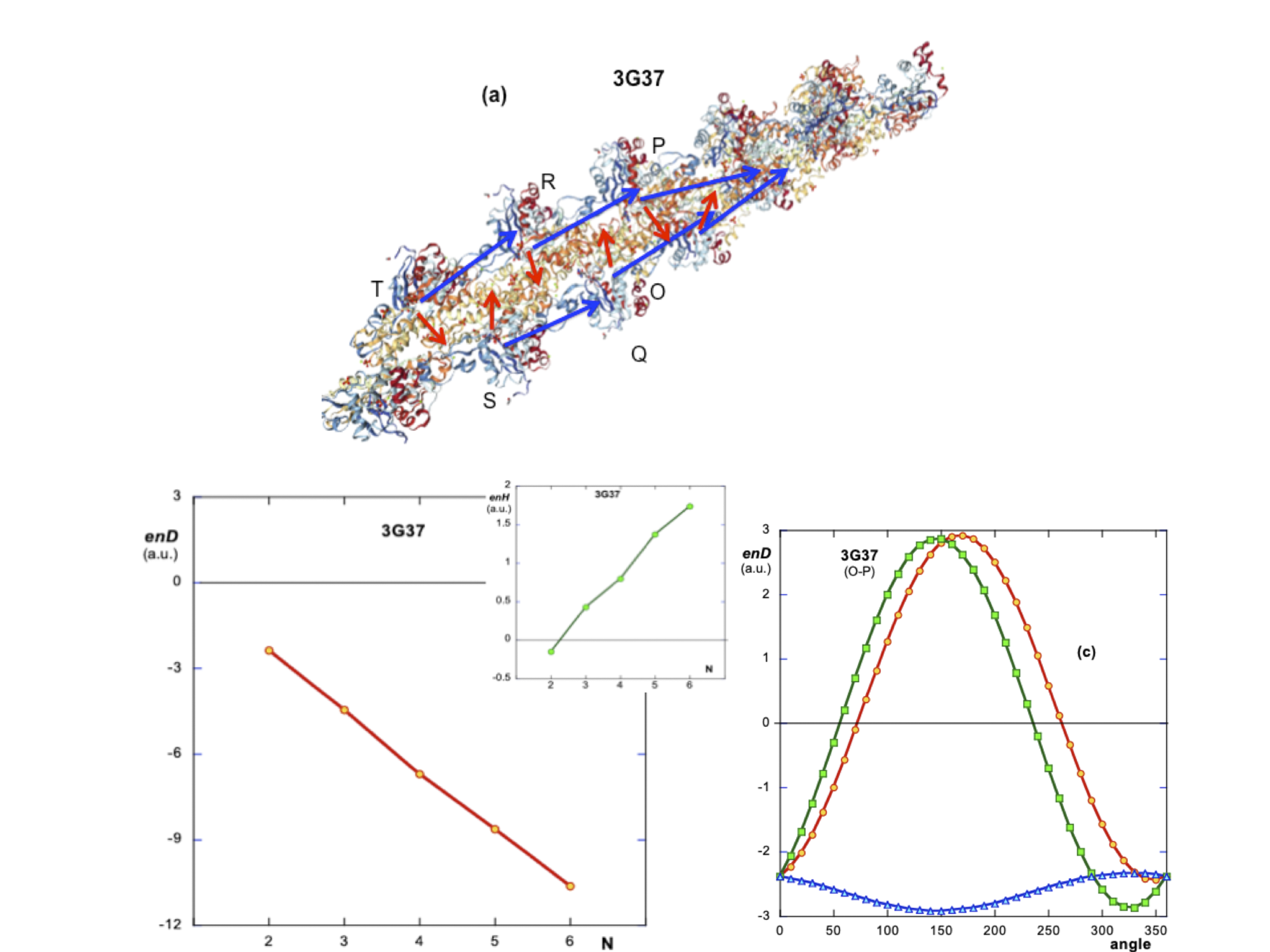

Supplement: S2 Fig — (a) Cryo-Electron structure of filamentous actin in the presence of phosphate according to Murakami et al., [24]. Blue and red arrows are H and D vectors respectively of some individual monomers in the polymer. Like in other actin polymers, H vectors tend to align with the polymer axis, while D vectors rotate perpendicularly to the axis of growth. (b) Averaging the energies of all the dimers reported: = –2.36 ± 0.055a.u. (±2.3%), the assembly is electrostatically driven. However, averaging enH energies of dimers a value of = –0.101 ± 0.021a.u. (±21%) is obtained. This means that dimers are drawn together by both electrostatic and hydrophobic forces. However, as seen in the inset of (b), from N ≥ 3, growth proceeds electrostatically only. (c) Energy distribution of enD when monomer P is rotated with respect to monomer O in the three directions of space. As in other cases, steric hindrances do not allow for perfectly symmetrical angular distributions. (TIF) [file pone.0216253.s003.tif]

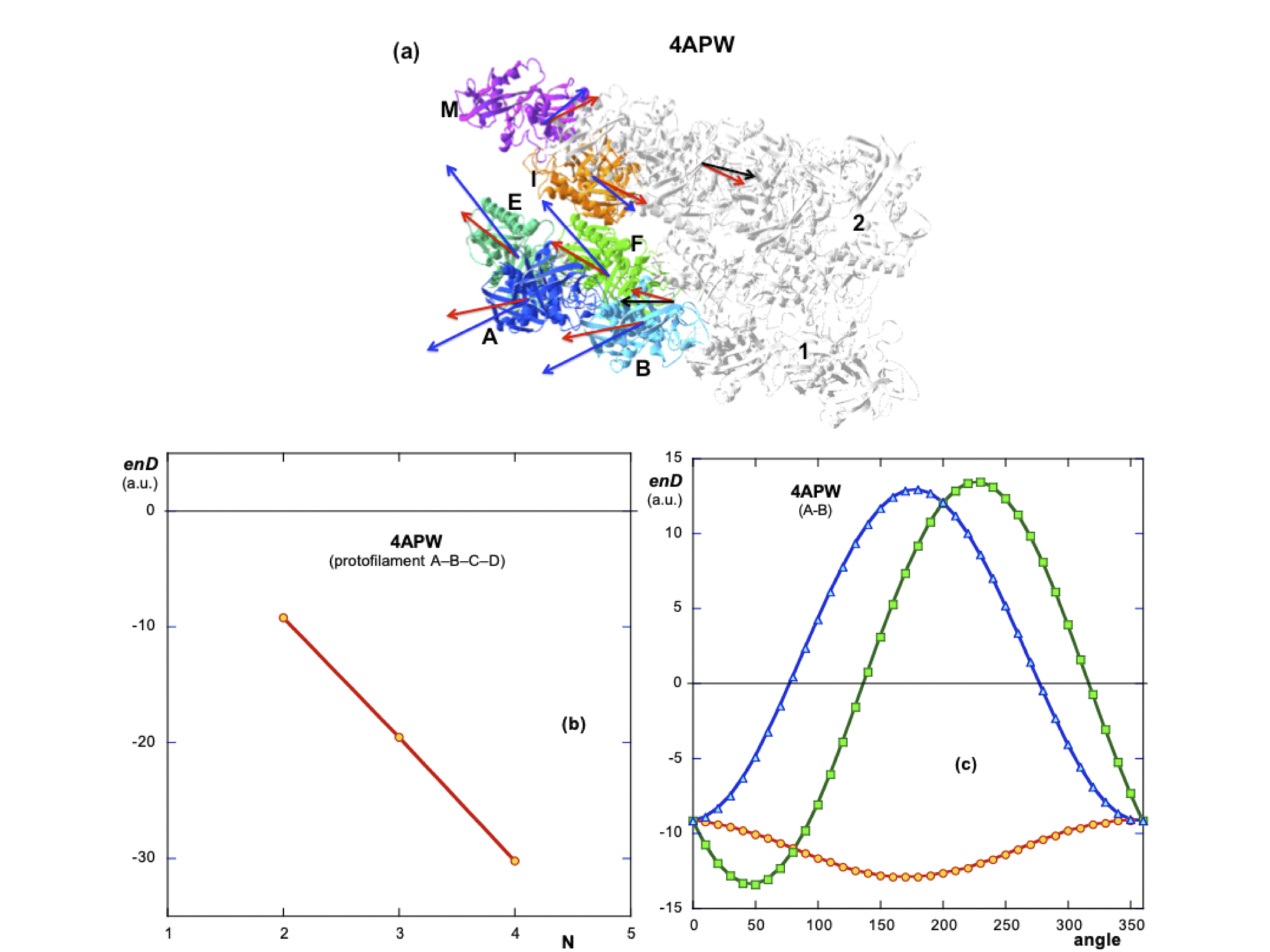

Supplement: S3 Fig — (a) A peculiar actin-like polymer described by Popp et al. [25], is formed by two strands (1–2). Blue and red arrows represent H and D vectors. Each strand is the result of the addition of protofilaments ABCD… and EFG…; (b) Formation of protofilament ABCD is formed electrostatically: = –9.79±0.37 a.u., = 0.213±0.039 a.u (±3.8% and ±18.3% error respectively). Protofilaments are added hydrophobically to form a strand. Although they do not appear in the Fig, strands are added in opposite directions electrostatically to form a fiber. (c) Rotation simulations of monomer B with respect to A. Near optimum enD for y-axis rotations (45°) and relative insensitive variation in the x-axis are due to steric limitations in the arrangement as seen in other systems. (TIF) [file pone.0216253.s004.tif]

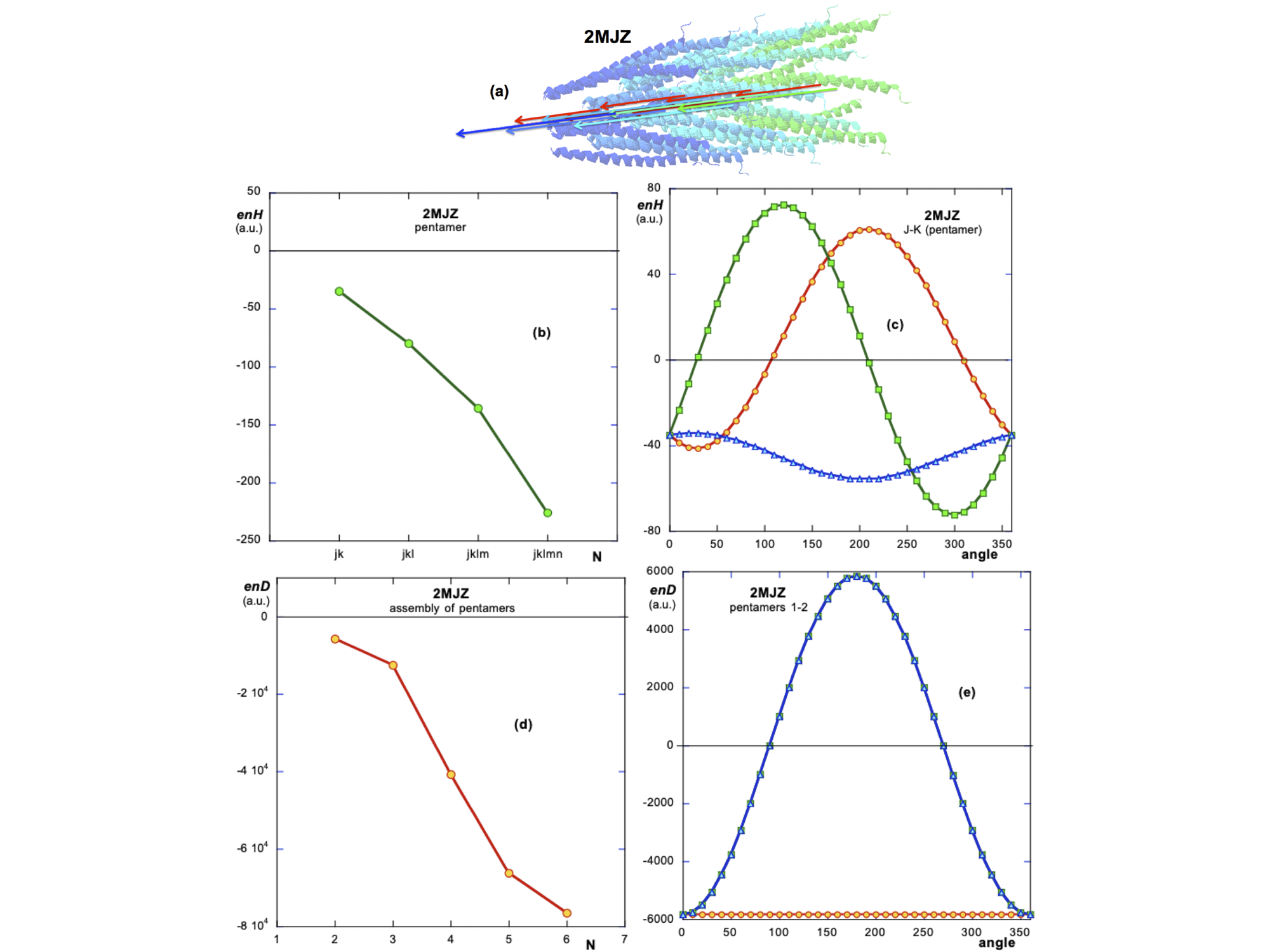

Supplement: S4 Fig — This structure was obtained combining magic-angle spinning NMR and Rosetta modeling [26]: Five α-helices associate as a close system to serve as an element of growth in a linear addition in this system. (a) Cartoon picture in which each bluish tone represents an added pentamer to the system. H vectors are drawn in colors matching their corresponding pentamers and D vectors are all colored in red. All these vectors have been drawn with a separation between them to better visualization although they are all coincident in direction. Computation of energy was carried out at two levels. First, the formation of the basic pentamer of each stage followed by the subsequent analysis of the addition of pentamers. (b) Variations of enD with N, number of elements in the assembly of one pentamer. The assembly of the pentamer is hydrophobically driven: = –43.6 ± 0.01a.u. (±0.01%); = 1860 ± 33.2.1a.u. (±1.8%). (c) Variations of enD under simulated rotations of the peptides forming each pentamer. Rotations around the y-axis show a minimum at –60° meaning that possible clashes prevent reaching the energy minimum. Rotations around the z-axis show a minimum at –160° although the energy variation is not as sensitive to rotations as in the other axis. (d) Variation of enH with N, number of pentamers added in the system as a whole. This addition is electrostatically driven. Simulation of rotations of a given pentamer with respect to its neighbor: = –11091 ± 4323a.u. (39%); = 249.9 ± 49.1a.u. (19.6%). (e) Variations of enH with rotated angle in the three direction of space. Given the symmetry present in this system, rotations around the x-axis leave enH of the system unaltered. The native value of enD is the optimal electrostatic energy. (TIF) [file pone.0216253.s005.tif]

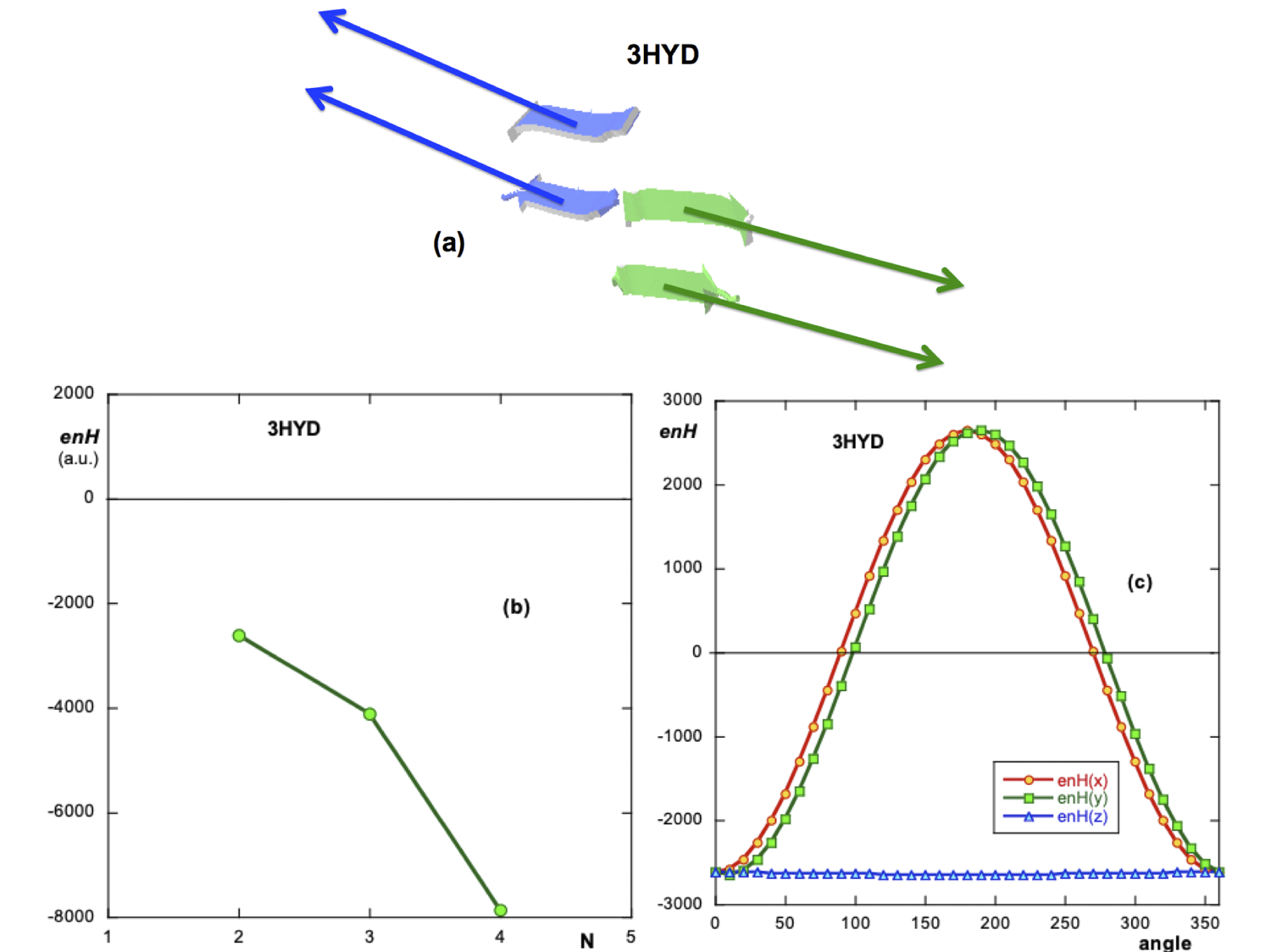

Supplement: S5 Fig — (a) Association of the LVEALYL fragment of insulin, leading to an amyloidosis-like self-assembly (PDBid 3HYD) as reported by Ivanova et al. [27]. This is a very simple system since no electric dipole moments are present in this system.(b) Quasi-linear variation of enH with N, number of assembled elements. The system is hydrophobically driven: = –2621.2 ± 13.5 a.u (±0.5% standard error). (c) enH variations under rotation simulations clearly show that the native angle (0°) formed by two adjacent monomers, i.e. two blue arrows or two green arrows in (a), leads to the minimum of enH with very little restrictions. (TIF) [file pone.0216253.s006.tif]

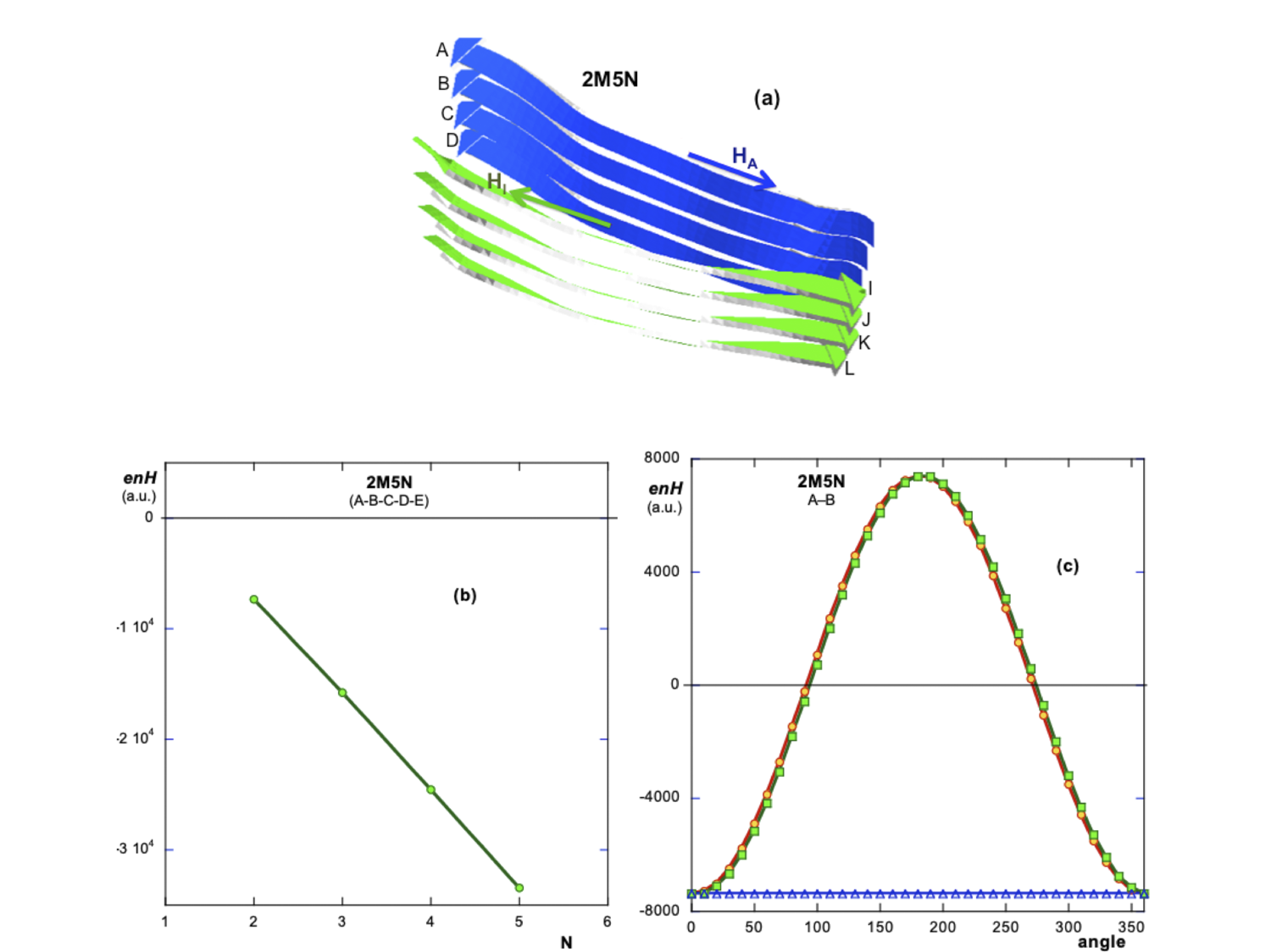

Supplement: S6 Fig — (a) Assembly of the basic YTIAALLSPYS peptide (PDBid 2M5N) related to Alzheimer disease [28]. This peptide also serves for the formation of other more complex filaments. As in the case of PDBid 3HYD, the absence of electrostatic negatively charged amino acids makes this growth particularly illustrative as an example of the membrane model since it does not include electric dipole moment. The system is hydrophobically driven: = –7453 ± 585.2a.u (±7.8% error); (b) And (c) follow the same codes as in former Figs. Note that a rotation of peptide B with respect to A around both x- and y-axis are quasi identical, whereas rotation around the z-axis leaves the energy unchanged since it is a rotation around its own axis. (TIF) [file pone.0216253.s007.tif]

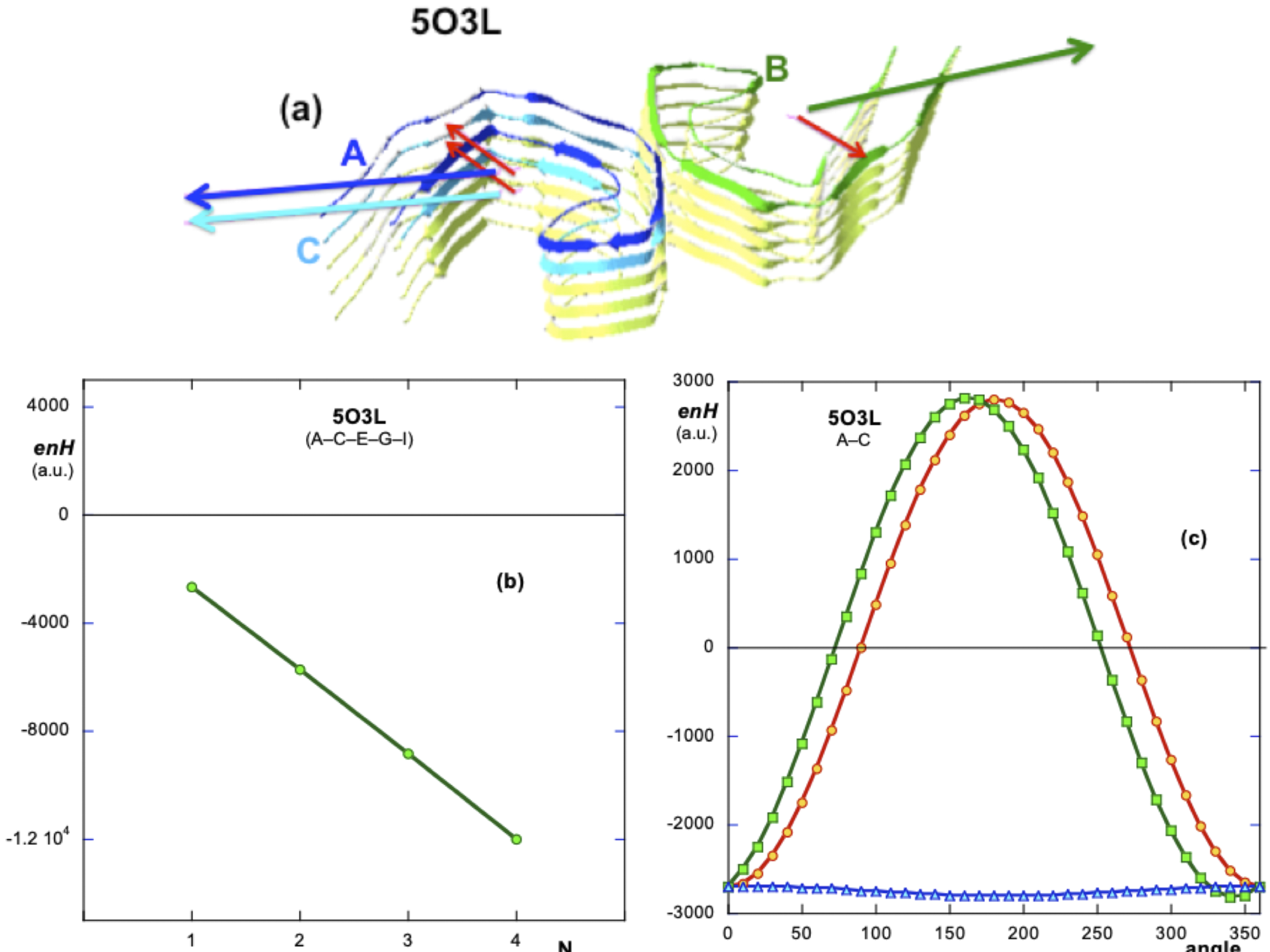

Supplement: S7 Fig — (a) Stacks of tau filaments in opposite orientations reported by Fitzpatrick et al. [29]. Blue and red arrows are H and D vectors respectively. (b) and (c) like in former Figs. The stacking is hydrophobically driven: = –0.302 ± 3.4x10^-6^a.u. (±0.001%); = 0.607 ± 5.5x10^-5^a.u. (±0.01%). Like in other similar cases, the monomeric peptides form stable stacks in a very symmetrical structure. These stacks form filaments by the dual joining of two stacks orienting their H vectors in opposite directions. (TIF) [file pone.0216253.s008.tif]

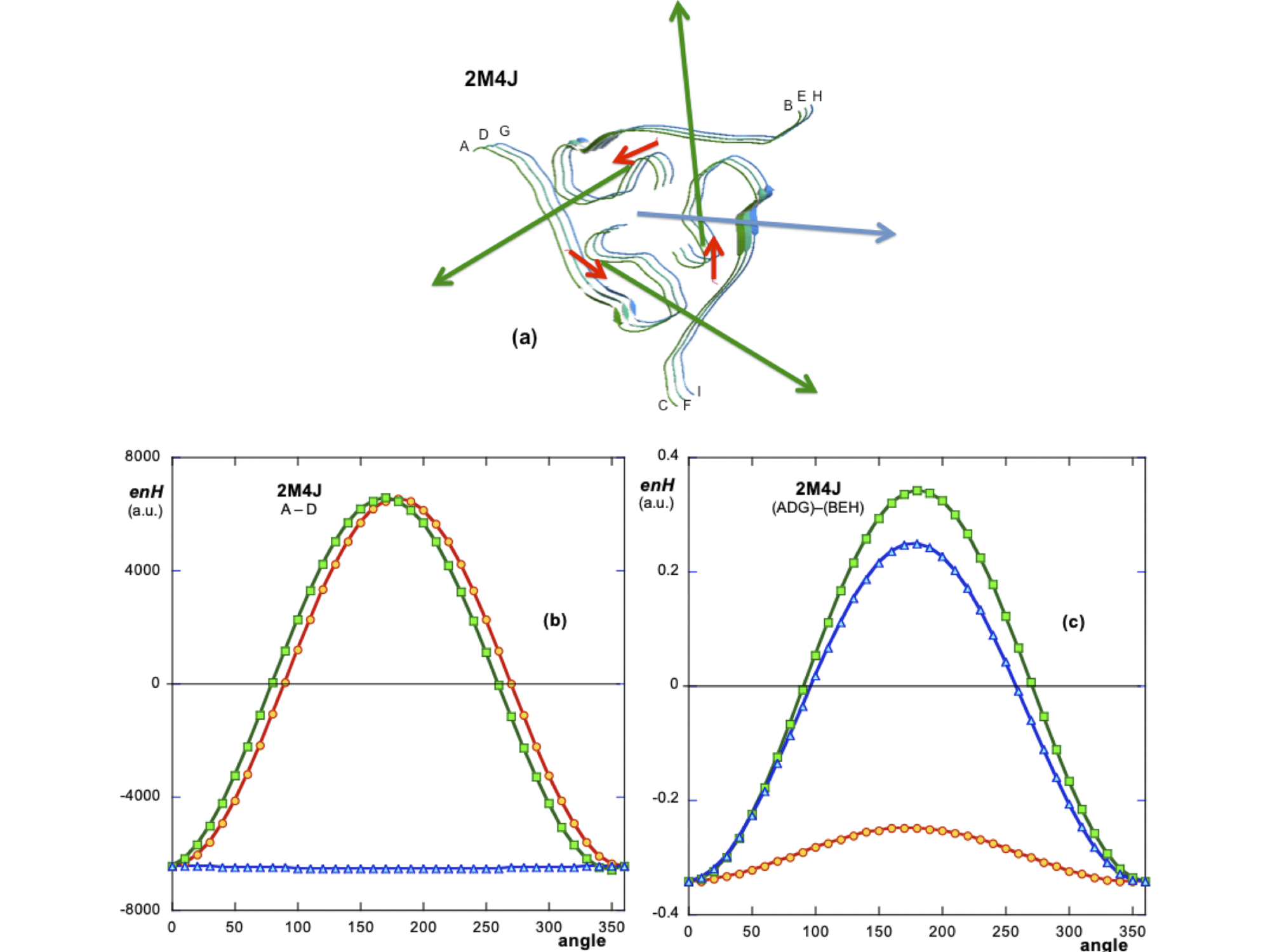

Supplement: S8 Fig — (a) Association of three stacks of Aß1–40 fibrils. Green and red arrows denote the individual H and D vectors of each single peptide. The assembly of each stack is hydrophobically driven: = –6863 ± 405.2a.u; = 3634 ± 213.2a.u (±5.9% and ±5.8% error respectively). (b) Simulation of rotations of peptide D with respect to peptide A. (c) Simulation of rotations of the stack ADG with respect to stack BEH. As can be seen, the association of stacks is also hydrophobic. The quasi-perfect symmetry of these energy curves reflects the perfect geometrical arrangement of this association. (TIF) [file pone.0216253.s009.tif]

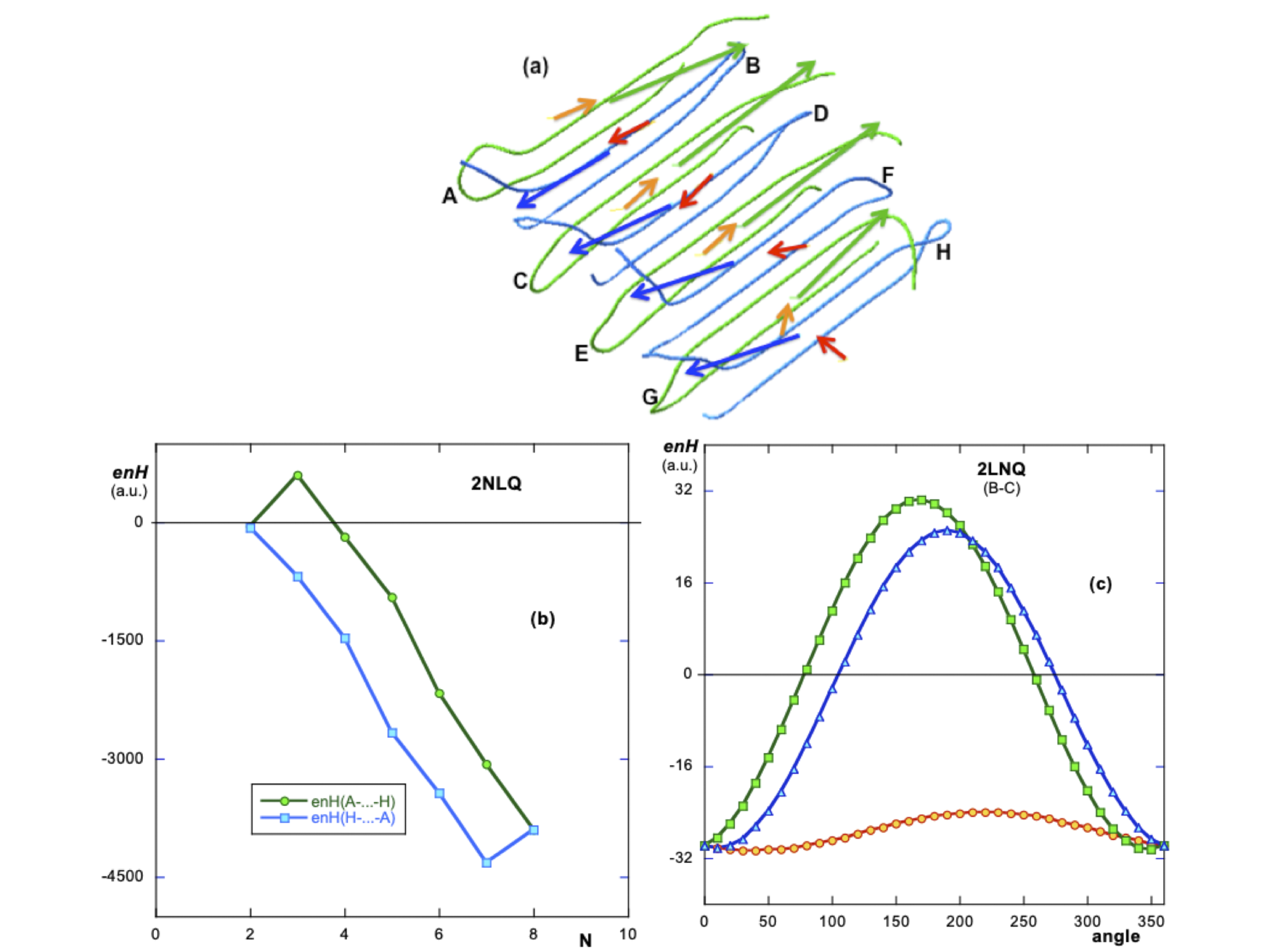

Supplement: S9 Fig — (a) Antiparallel array of ß-amyloid fragments (with D23N mutation) as reported by Qiang et al. [31]. (b) Variation of energy enH of the assembly as a function of N, number of elements. It was found that this assembly is hydrophobically driven: = –55.56 ± 4.30 a.u. (±7.7%); = 2040 ± 974 a.u. (±47.7%) for all the dimers computed. Certain variability in the shape and relative orientation of the individual peptides is observed in (a). This translates in a concomitant variability in the relative magnitude and direction of their individual H and D vectors. It was found necessary to compute the total energy enH following the sequence order A–B–C–D–E–F–G–H (green circles) and compare the result with the total enH energy following the reverse order (blue squares). Note that in the first case, for N = 3, enH shows a positive (repulsion) value. This is attributed to a certain “noise” originated by the lack of perfect regularity in the arrangement. This abnormality changes its position when the energy growth is computed backwards, that is, H-G-F-… (c) The angular distributions of enH when monomer C is rotated vs. monomer B, show optimal energies very near to the native configuration. (TIF) [file pone.0216253.s010.tif]

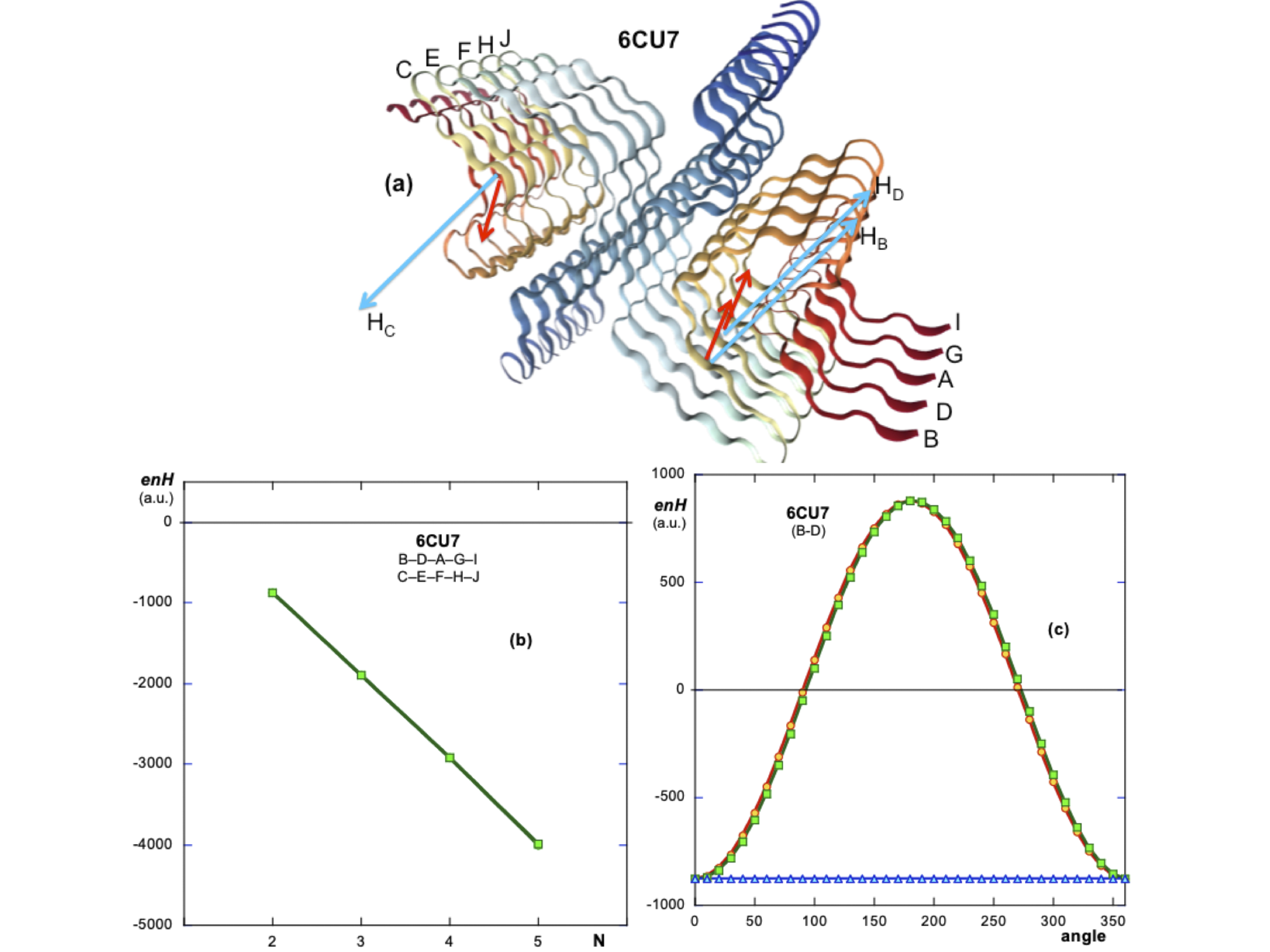

Supplement: S10 Fig — (a) Set of two stacks of α-synuclein peptides facing each other (source: PDB homepage) as described by Li et al. [32]. Blue arrows represent H vectors of the first elements in the stack and red arrows their D vectors. This assembly is hydrophobically driven: = –894.6 ± 6.3a.u. (±0.7%); = 1008.5 ± 4.4a.u. (±0.4%). e. (b) Variation of enH with the number of elements in both stacks, N. These energies were computed by adding the elements following this sequence B-D-A-G-I on one hand and C-E-F-H-J on the other hand. Both stacks attract each other by hydrophobic energy (data not shown). (c) Effect of rotations of H vector of element D with respect to that of element B. It can be seen that hydrophobic energy holds the system assembled at the optimum orientation as is in most cases of perfect geometrical arrangement. (TIF) [file pone.0216253.s011.tif]

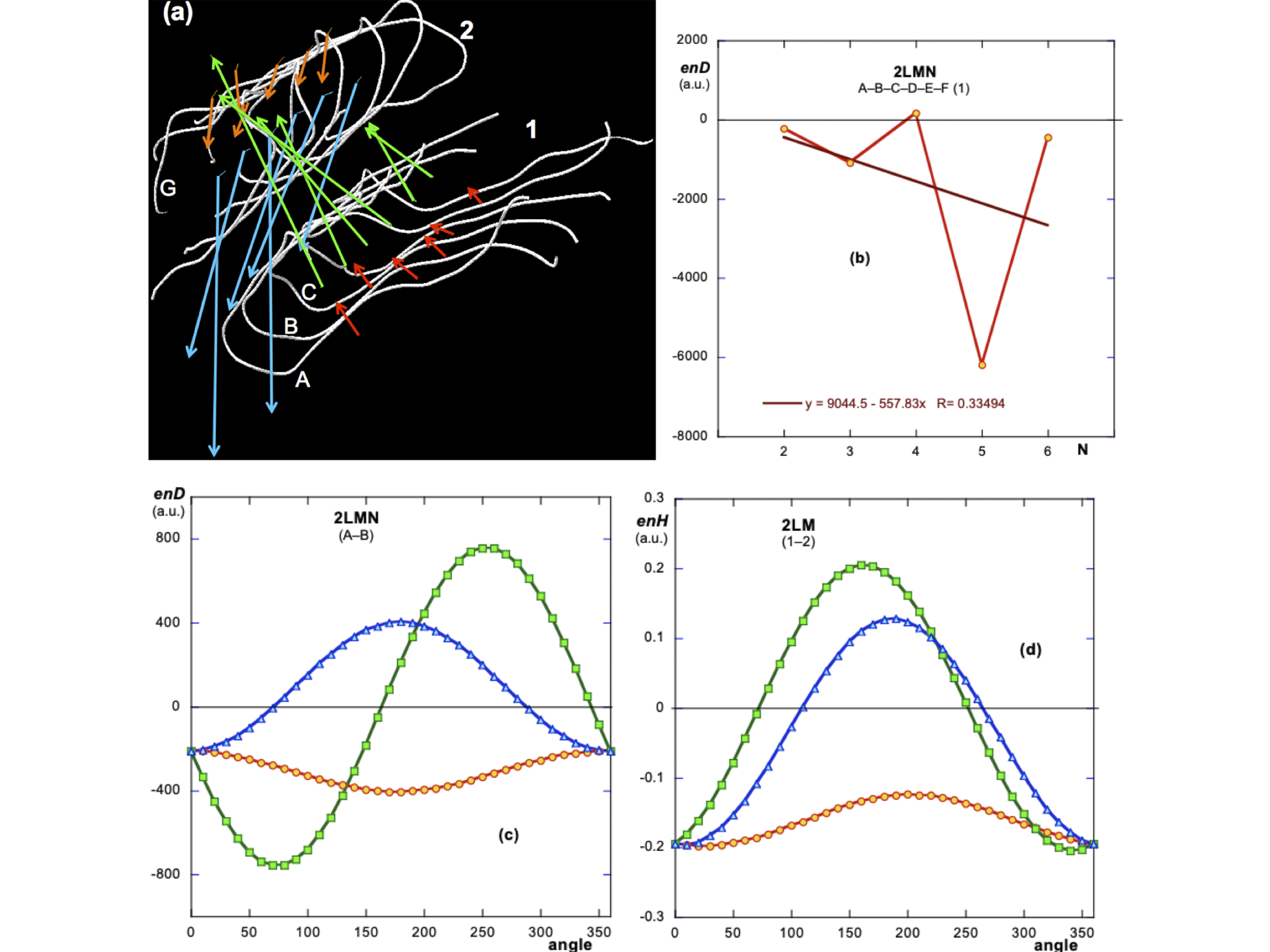

Supplement: S11 Fig — (a) 2 stacks of beta-amyloid fibrils in opposite orientations (1 and 2) reported by Paravastu et al. [33]. Green and blue arrows represent the H vectors of the individual peptides. Red and orange vectors, their D vectors. The energy that maintains each stack is electrostatic: = –663.9 ± 159.0a.u. (±24.0%); = 694.9 ± 234.1a.u. (±33.7%). (b) Variation of enH with N corresponding to stack 1. Stack 2 shows a similar level of variability or noise that is attributable to the variability in shape and relative orientation of the single monomers. (c) Angular variation of enH and enD under rotation of element B with respect to element A. The variability of orientations of the H and D vectors shown in the assembly (±30°) is reflected in (c) by the fact that peptides A and B are not set at their optimal energy, especially for rotations in the y-axis. (d) The energy linking both stacks is hydrophobic. (TIF) [file pone.0216253.s012.tif]

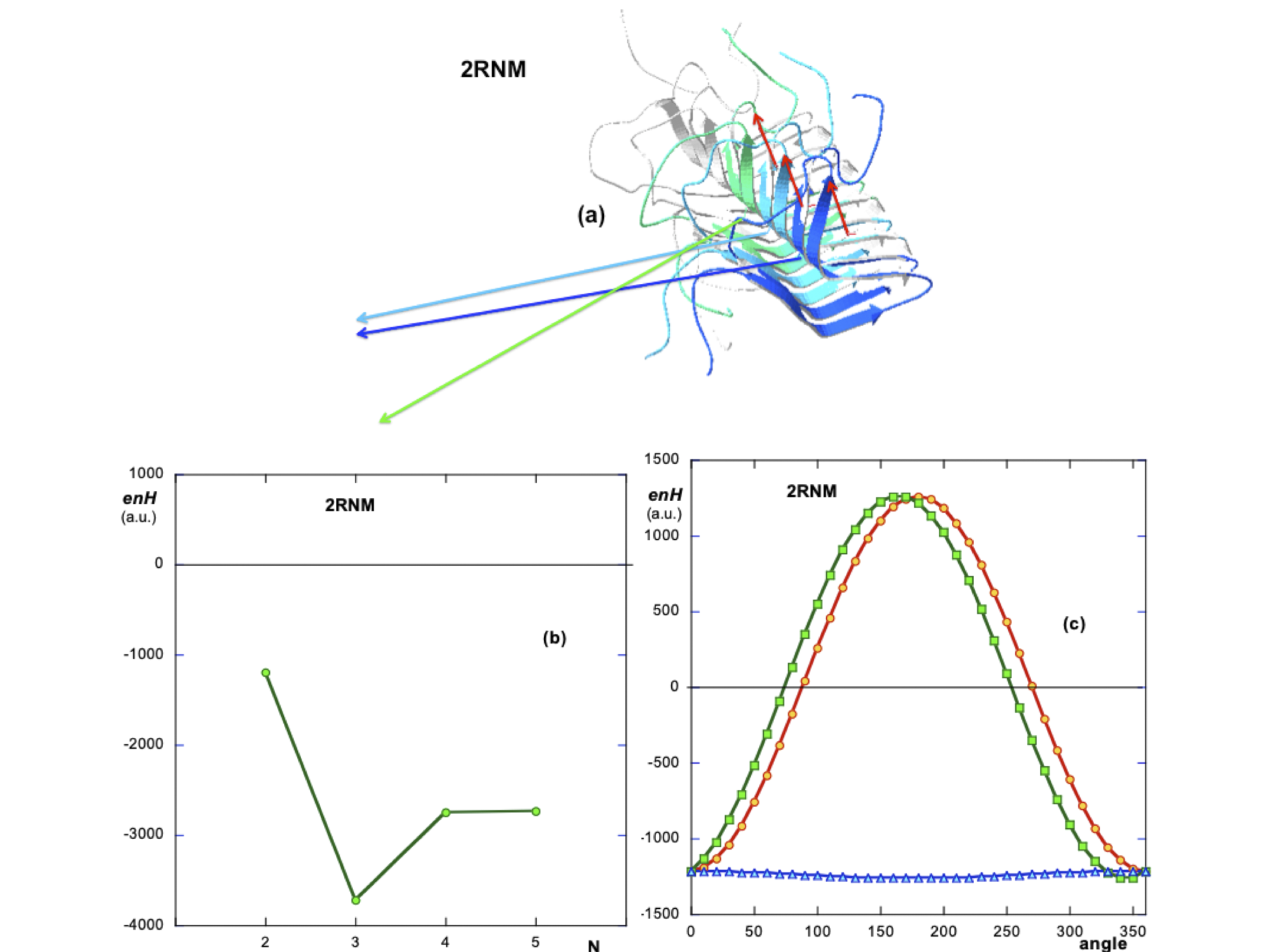

Supplement: S12 Fig — (a) Prion fibrils arranged parallel according to Wasmer et al. [34]. Arrows in cold colors: individual H vectors of the peptides. Red arrows: D vectors. (b) and (c) Variations like in former Figs. Due to the unstructured tails displayed by each peptide, and the concomitant dispersion in the magnitude and direction of their H vectors, the system is noisy in the sense of having a notable variability in its enH values: = –680.0 ± 340.7a.u. (±50%); = 295.8 ± 39.6 a.u. (±13.4%). It is interesting to note here that this stacked structure can be associated with other similar stacks as described by Smaoui et al. [35]. These authors show that these filaments are formed by three associated stacks arranged at a relative orientation of 120° with respect to each other (see PDBid 2M4J). (TIF) [file pone.0216253.s013.tif]

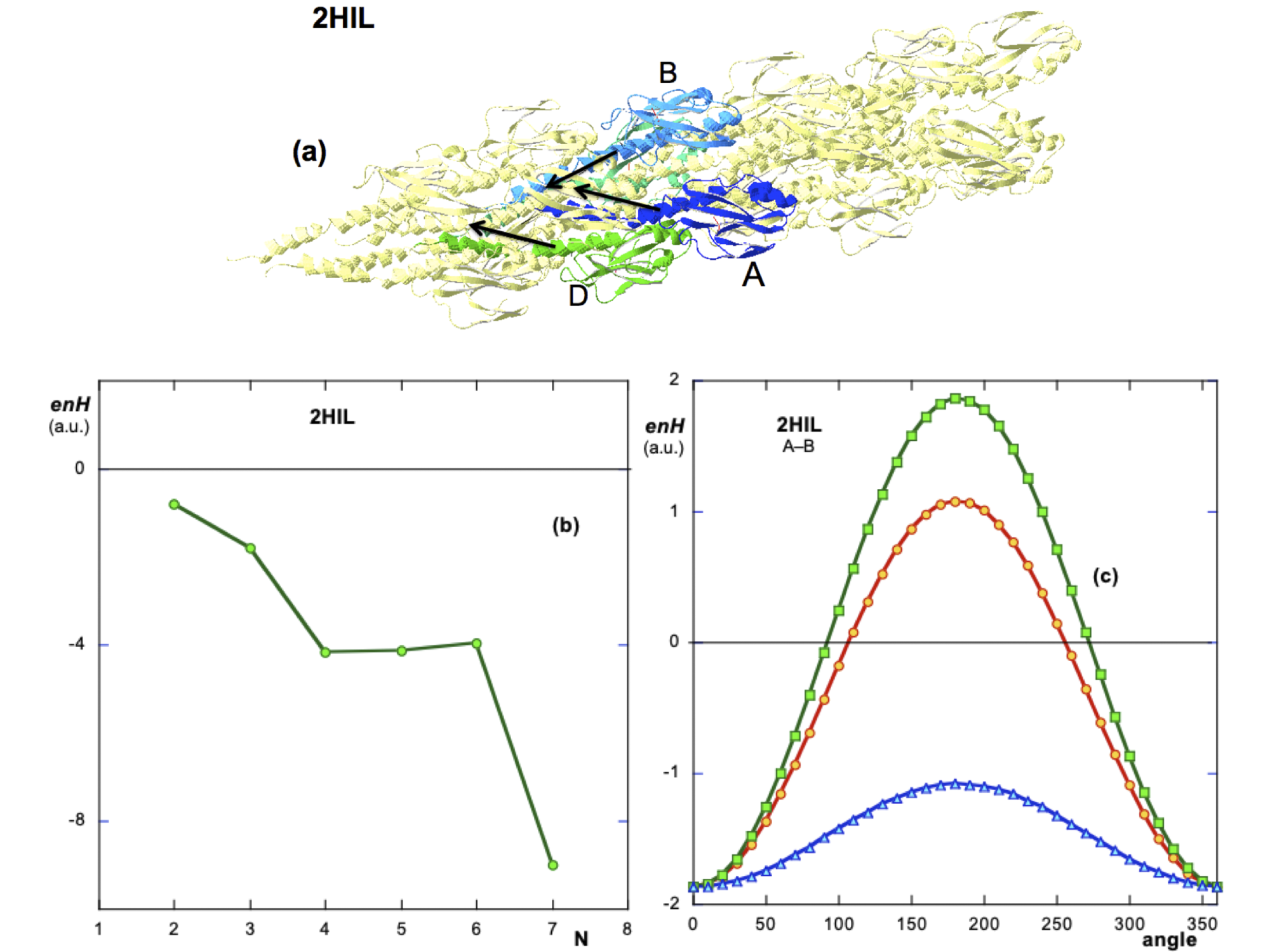

Supplement: S13 Fig — (a) Spiraling assembly by three-helix bundles from type IV pili of inner membrane bacteria as described by Craig et al. [36], obtained from Cryo-Electron Microscopy experiments. Some H vectors (A, B and D subunits) are depicted (black arrows). (b) The variation of energy enH with N shows that the assembling of this system is hydrophobically driven: = –0.704 ± 0.079a.u. (±11.2%); = 0.204 ± 0.006a.u. (±2.9%). (c) Angular distribution of energies when subunit B is rotated with respect to subunit A, showing optimal orientation from perfectly symmetrical energy distributions. (TIF) [file pone.0216253.s014.tif]

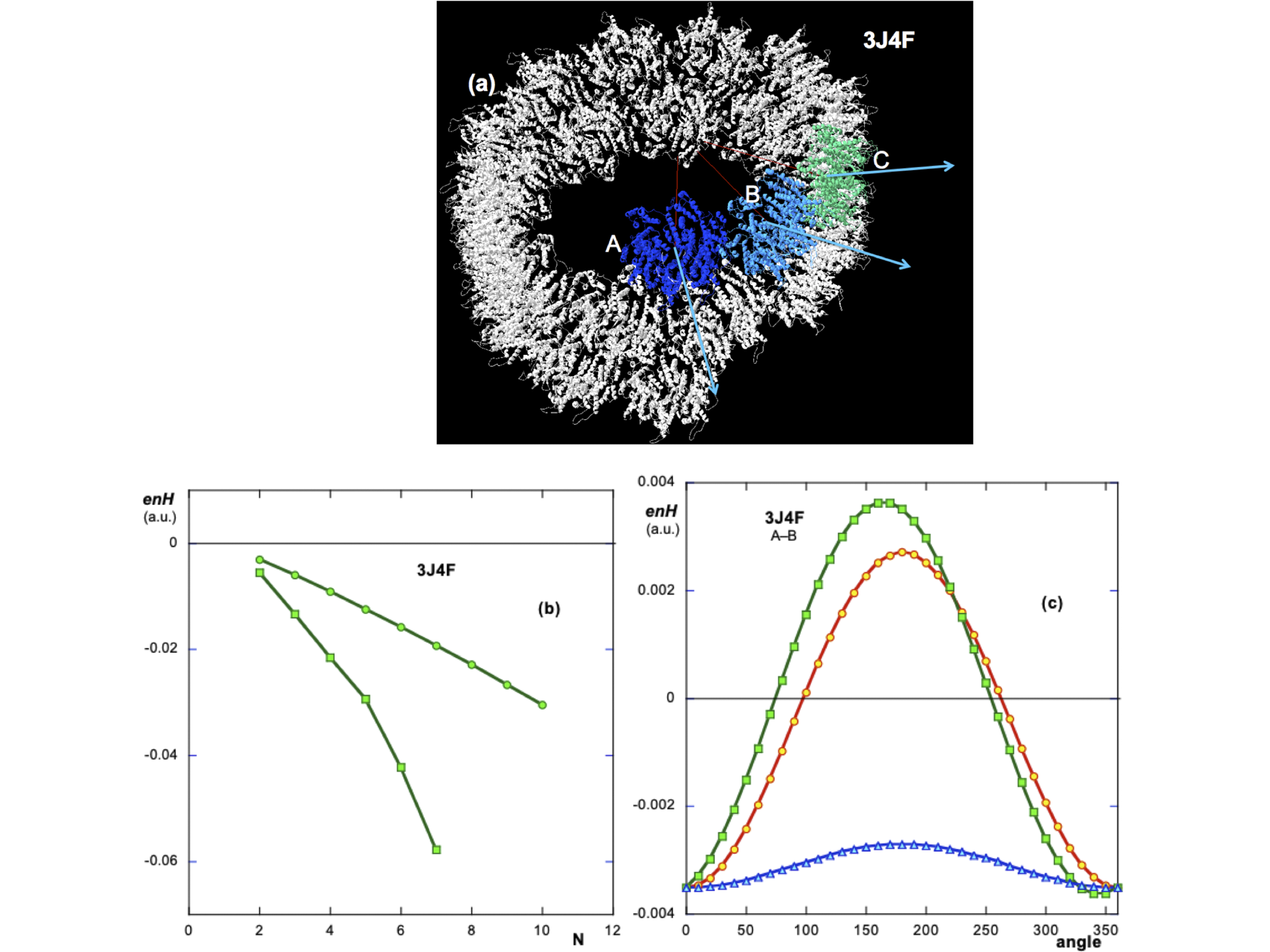

Supplement: S14 Fig — (a) Protein capsid of HIV-1 virus, reported by Zhao et al. [37]. The basic element of growth of this system is a hexamer. The first three hexamers (A-B-C) have been colored and their H vectors are displayed as pale blue arrows. (b) Variations of enH for the growing assembly (circles) or growth around an inner element, 13 (squares). The system is hydrophobically driven: = –(2.3 ± 0.25) x10-3a.u. (±10.8%); = 0.861 ± 0.087a.u. (±10.1%). (c) Simulation of rotations of element B with respect to element A. The configuration of this system is very close at its optimal energy. (TIF) [file pone.0216253.s015.tif]

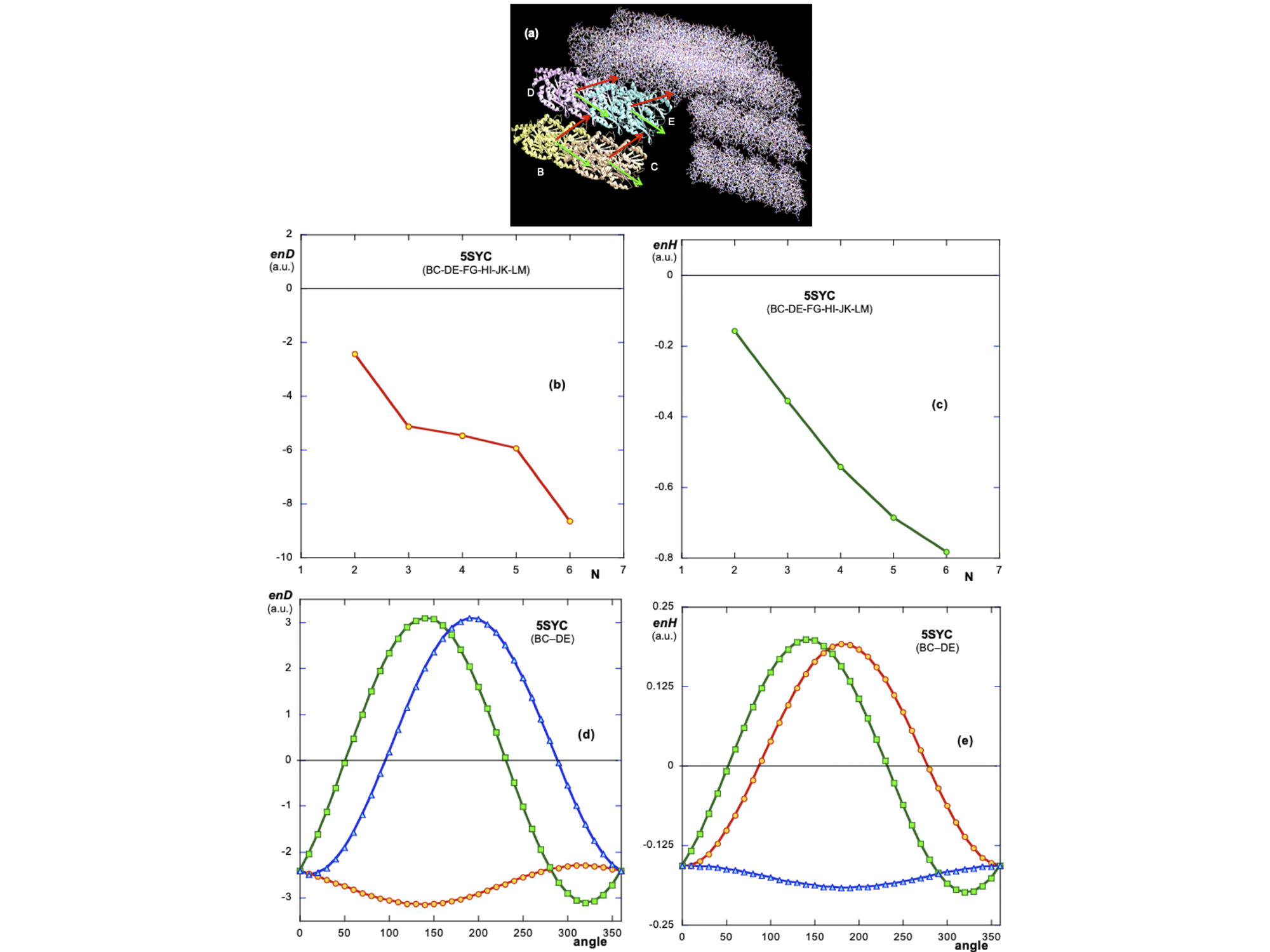

Supplement: S15 Fig — (a) Growing microtubule reported by Kellogg et al. [38]. The first four elements (tubulin dimers) have been cultured for clarity. Green and red arrows represent H and D vectors respectively. Although the basic element in this system is a tubulin heterodimer (i.e. yellow in (a)), according to these authors, the growth proceeds by the addition of a dimer of dimers (i.e. yellow and orange). This is the only system found in which both enH and enD are attractive energies: = –0.135 ± 0.018a.u. (±13.3%); = –1.475 ± 0.601a.u. (±41%). (b) and (c) plot the variations of enD and enH as new elements are added to the tubule. (d) and (e) Representations of rotations of D vectors (d) and H vectors (e) as described in Methods. Native energies are only near their optima especially those rotations around the y-axis. It is considered that this lack of symmetries in these distributions is responsible for the appearance of helicity, giving rise to the formation of the tubule as a result. (TIF) [file pone.0216253.s016.tif]

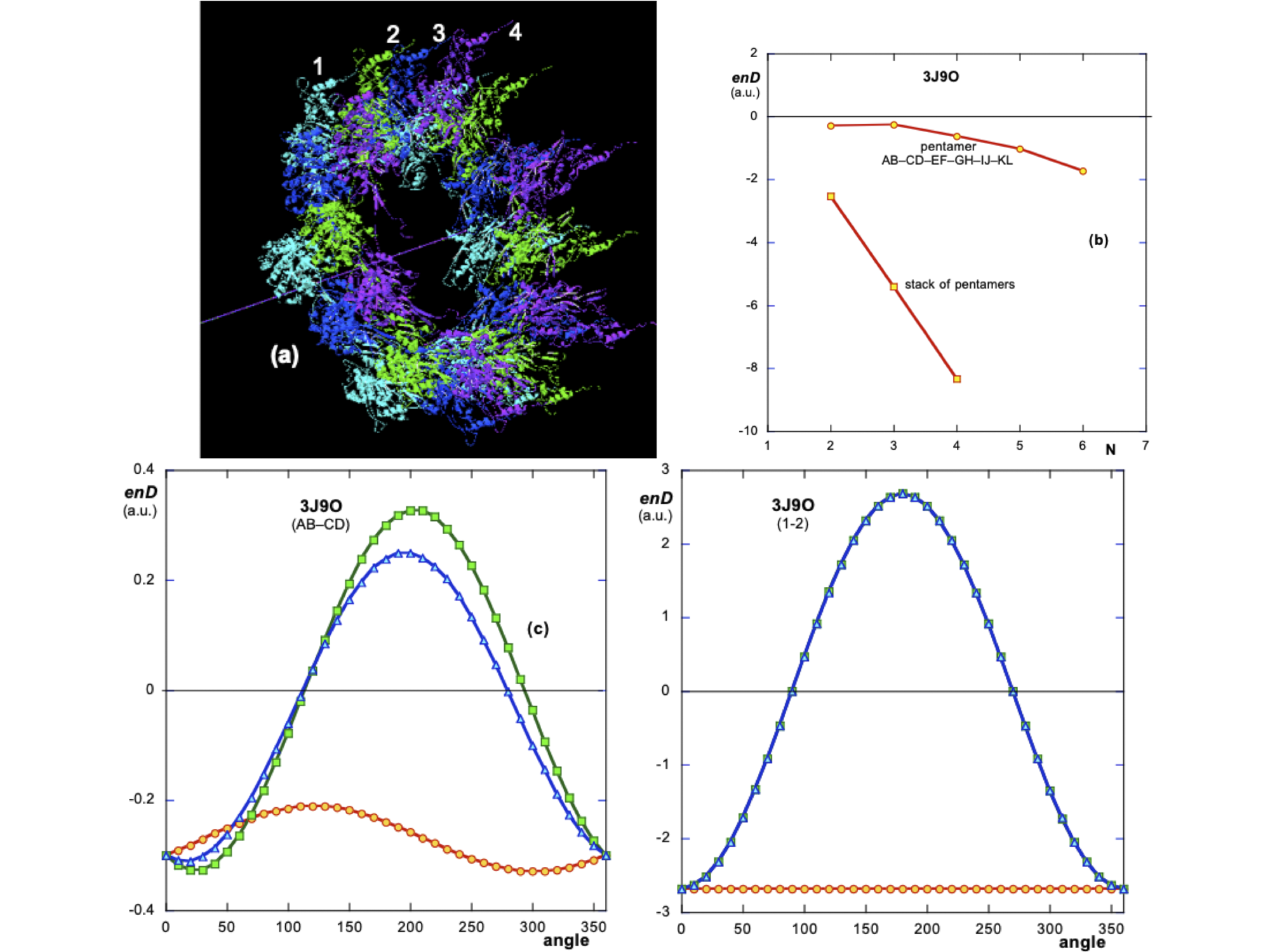

Supplement: S16 Fig — (a) Reconstruction of the structure of Type VI secretion system by Cryo-EM by Nazarov et al. [39]. It is composed by the stacking of rings each formed by six heterodimers and resembling (each ring) a crown. The heterodimers are designed as “AB”, “CD”, etc. The stacking proceeds by rotating the next ring 60° around the axis of growth with respect to the previous one. (a) shows four of these rings: (1) pale blue, (2) dark blue, (3) green and (4) purple. Thin lines design the direction of H and D vectors and the axis growth as well. The formation of the ring is electrostatic: = –2.532 ± 0.158a.u. (±34.5%); = 0.159 ± 0.040a.u. (±25.1%) The stacking of rings is also electrostatic: = –0.223 ± 0.077a.u. (±6.2%); = 0.187 ± 0.014a.u. (±7.5%). (b) Double plot of the variation of electrostatic energy enD vs. N in the formation of one of the rings (circles) and the stacking (squares). (c) and (d) Plots of the variations of enD for the ring formation and the stacking of rings respectively. (TIF) [file pone.0216253.s017.tif]
